# Selective Azapeptide CD36 Ligand MPE-298 Regulates oxLDL-LOX-1-Mediated Inflammation and Mitochondrial Oxidative Stress in Macrophages

**DOI:** 10.3390/cells14050385

**Published:** 2025-03-06

**Authors:** Mukandila Mulumba, Catherine Le, Emmanuelle Schelsohn, Yoon Namkung, Stéphane A. Laporte, Maria Febbraio, Marc J. Servant, Sylvain Chemtob, William D. Lubell, Sylvie Marleau, Huy Ong

**Affiliations:** 1 Faculté de Pharmacie, Université de Montréal, Montréal, QC H3C 3J7, Canada; mukandila.mulumba@umontreal.ca (M.M.); catherine.le.13@umontreal.ca (C.L.); marc.servant@umontreal.ca (M.J.S.); sylvie.marleau@umontreal.ca (S.M.); 2 Institut des Sciences Pharmaceutiques de Suisse Occidentale (ISPSO), Section Sciences Pharmaceutiques, Département des Sciences, Université de Genève, 1205 Genève, Switzerland; e.schelsohn@gmail.com; 3 Department of Medicine, McGill University, Montreal, QC H4A 3J1, Canada; yoon.namkung@mcgill.ca (Y.N.); stephane.laporte@mcgill.ca (S.A.L.); 4 Department of Dentistry, University of Alberta, Edmonton, AB T6G 2H5, Canada; febbraio@ualberta.ca; 5 Faculté de Médecine, Centre Hospitalier Universitaire Sainte-Justine, Montréal, QC H3T 1C5, Canada; sylvain.chemtob@umontreal.ca; 6 Département de Chimie, Université de Montréal, Montréal, QC H3C 3J7, Canada; william.lubell@umontreal.ca

**Keywords:** MPE-298, CD36 synthetic azapeptide ligand, inflammation, oxidative stress, mitochondrial damage, LOX-1, macrophages

## Abstract

Macrophage mitochondrial dysfunction, caused by oxidative stress, has been proposed as an essential event in the progression of chronic inflammation diseases, such as atherosclerosis. The cluster of differentiation-36 (CD36) and lectin-like oxLDL receptor-1 (LOX-1) scavenger receptors mediate macrophage uptake of oxidized low-density lipoprotein (oxLDL), which contributes to mitochondrial dysfunction by sustained production of mitochondrial reactive oxygen species (mtROS), as well as membrane depolarization. In the present study, the antioxidant mechanisms of action of the selective synthetic azapeptide CD36 ligand MPE-298 have been revealed. After binding to CD36, MPE-298 was rapidly internalized by and simultaneously induced CD36 endocytosis through activation of the Lyn and Syk (spleen) tyrosine kinases. Within this internalized complex, MPE-298 inhibited oxLDL/LOX-1-induced chemokine ligand 2 (CCL2) secretion, abolished the production of mtROS, and prevented mitochondrial membrane potential depolarization in macrophages. This occurred through the inhibition of the multiple-component enzyme nicotinamide adenine dinucleotide phosphate (NADPH) oxidase 2 (NOX2) by oxLDL-activated LOX-1, which was further supported by the reduced recruitment of the p47phox subunit and small GTPase (Rac) 1/2/3 into the plasma membrane. A new mechanism for alleviating oxLDL-induced oxidative stress and inflammation in macrophages is highlighted using the CD36 ligand MPE-298.

## 1. Introduction

Atherosclerosis is a chronic inflammatory disease that significantly increases cardiovascular mortality risk. In its early stages, macrophages accumulate in the subendothelial space of the arterial walls, primarily ingesting oxidized low-density lipoprotein (oxLDL), which triggers the disease. Excessive oxLDL in macrophages disrupts cholesterol metabolism, causing mitochondrial oxidative stress and inflammation, leading to foam-cell formation and unstable plaques. Playing a critical role in the early manifestation of atherosclerosis, the cluster of differentiation 36 receptor (CD36) binds and facilitates oxLDL uptake by macrophages through the formation of a heteromeric complex consisting of tetraspanin CD9, integrins β1/2 and the adaptor receptor FcRγ [1,2]. The low-density lipoprotein receptor-1 (LOX-1), the main endocytic receptor of oxLDL in endothelial cells, also facilitates oxLDL uptake in macrophages [3], causing oxidative stress in the mitochondria via mitochondrial DNA damage and the generation of mitochondrial reactive oxygen species (mtROS). The interaction between LOX-1 and CD36 enhances the uptake of oxLDL by macrophages and foam cell formation [4].

The CD36 heteromeric complex present on the membrane surface of macrophages also interacts with Toll-like receptor 2/6 (TLR2/6) heterodimers [5,6] and sodium–potassium adenosine triphosphatase (Na/K ATPase) to induce cellular responses and to drive inflammation [1,2,7]. In addition, CD36 on the plasma membrane could also commence a signaling cascade upon oxLDL binding by initiating a non-transcriptional endocytic pathway featuring recruitment and activation of Src non-receptor tyrosine kinases, such as Lyn and spleen tyrosine kinases (Syk), and actin polymerization, which results in oxLDL accumulation and formation of cholesterol crystals in lysosomes, causing functional impairment [1,2,8]. In addition to being the endogenous CD36 ligand, the binding of oxLDL to LOX-1 facilitates its endocytosis into macrophage and endothelial cells, leading to oxidative stress, as well as the activation of nuclear factor kappa B (NF-kB) [3,9]. The signaling pathway controlling LOX-1-mediated ROS generation involves the activation of nicotinamide adenine dinucleotide phosphate (NADPH) oxidase 2 (NOX2) and c-Jun N-terminal kinase (JNK) [10,11,12,13].

Previously, we reported that CD36-selective growth-hormone-releasing peptide (GHRP) 6 derivatives, including MPE-298, reduced efficient macrophage recruitment to aortic lesions [14], dampened inflammation [15,16], and arrested lesion progression in apolipoprotein E (ApoE)-deficient mice fed a high-fat, high-cholesterol diet [14,15]. In a retinal inflammatory mouse model induced by photo-oxidative stress, MPE-001, an azapeptide derivative of GHRP-6, reduced mononuclear phagocyte accumulation at the subretinal level and preserved the integrity of retinal photoreceptor layers [16]. Treatment with MPE-001 protected retinal pigment epithelial cells from mtROS which was induced by oxidative stress. Moreover, MPE-001 promoted macrophage metabolic reprogramming from an M1 toward an M0 phenotype, as characterized by the increases in the mitochondrial electron transport chain complex subunits and oxygen consumption rate [16]. Recently, cyclic azapeptide derivative MPE-298, a cyclic semicarbazide GHRP-6 derivative that exhibits superior binding affinity to CD36 [17,18], reduced systemic inflammation, apoptosis, and lipid-laden macrophages in aortic lesions [15]. The mechanisms underlying the CD36-mediated MPE-298 protective effect against mitochondrial damage have yet to be deciphered.

In the present study, a bioluminescence resonance energy transfer (BRET) approach was developed to examine the mechanisms of MPE-298/CD36 internalization in J774A.1 and RAW264.7 macrophage-like cells. Internalization of the MPE-298/CD36 complex was found to be essential for modulating oxLDL-triggered mitochondrial stress and inflammation, by blocking oxLDL-LOX-1 signaling and preventing NOX2 activation. Consequently, the endocytosis of MPE-298/CD36 complex resulted in the disruption of the translocation of JNK and p66Shc into mitochondria with inhibitory effect on mitochondrial ROS production.

## 2. Materials and Methods

### 2.1. Reagents

Prolume Purple (Methoxy e-CTZ) was purchased from NanoLight Technology^TM^ (Pinetop, AZ, USA); the pCMV3-mCD36 C-GFPSpark tag for mCD36 expression from Sino Biologicals Inc. (Beijing, China); TransIT-X2^®^ Dynamic Delivery System from Mirus Bio LCC (Madison, WI, USA); Alexa-fluor^®^594 anti-rabbit IgG, Clone 6B9G9, isotype Mouse IgG1κ, macrophage colony-stimulating factor (rmM-CSF), and interferon gamma (IFNγ) all from BioLegend (San Diego, CA, USA); cytochalasin D (actin polymerization inhibitor), PP1, PP2 (Src inhibitors), piceatannol (Syk kinase inhibitor), ATTO-465 N-hydroxysuccinimidyl-esters (NHS), and *E. coli* (0111:B4) lipopolysaccharide (LPS) all from Sigma-Millipore (St. Louis, MO, USA); MitoSox-Red, Hoechst-33342, 5,5′,6,6′-tetrachloro-1,1′,3,3′-tetraethylbenzimidazolylcarbocyanine iodide (JC-1), CellLight^TM^ Late Endosomes-RFP, and CellLight^TM^ Lysosomes-RFP all from Invitrogen-Life Technologies (Carlsbad, CA, USA); and inhibitors 2-bromohexadecanoic acid (2-bromopalmitate, inhibitor of depalmitoylation), sulfosuccinimidyl oleate (SSO, CD36 inhibitor), and BI-0115 (LOX-1 inhibitor) from AbMole BioScience (Houston, TX, USA), Cayman Chemical (Ann Arbor, MI, USA), and MedChemExpress (Monmouth Junction, NJ, USA), respectively.

### 2.2. Antibodies

Antibodies against early endosome antigen 1 (EEA1), Rab7, Rab11, lysosome-associated membrane protein (Lamp) 1, Rac1/2/3, phospho-Tyr416 and total Src family kinases, phospho-Tyr397 and total Lyn, JNK and phospho-(Thr183/Tyr185) JNK, all were purchased from Cell Signaling (Danvers, MA, USA). SHC-adaptor protein 1 (Shc1) was from R&D System (Minneapolis, MN, USA). p47phox was from Novus Biologicals (Minneapolis, MN, USA). Total spleen tyrosine kinase (Syk) and phospho-Tyr348 Syk were from Invitrogen-Life Technologies (Carlsbad, CA, USA) and Cusabio (Houston, TX, USA), respectively.

### 2.3. CD36 Subcellular Localization and Immunofluorescence

Murine cell line J774A.1 (ATCC, Manassas, VA, USA) macrophages (100,000 cells) were seeded overnight in Dulbecco’s Modified Eagle Medium (DMEM, Wisent Inc., Saint-Jean-Baptiste, QC, Canada) supplemented with 10% fetal bovine serum (FBS, Wisent Inc, Saint-Jean-Baptiste, QC, Canada) and 1% penicillin/streptomycin in 8-well glass chamber slides and grown at 37 °C in 5% CO_2_. The next day, cells were incubated with 1 μM of 5-azacytidine for 2 h. Cells were transiently transfected with the mCD36-GFPSpark plasmid using the TransIT-X2^®^ reagent following the manufacturer’s instructions. Briefly, 176 ng of cDNA mixture containing pcDNA3.1/CD36-GFPSpark plasmids (2.5:1 ratio) were mixed with TransIT-X2^®^ reagent (ratio of 1 μg of cDNA to 4 μL of the reagent) for 20 min to allow the formation of cDNA/reagent complex. The mixture was added into the well containing cells in DMEM supplemented with 10% FBS, 0.1% penicillin/streptomycin and 1 μM of 5-azacytidine, and incubated at 37 °C in 5% CO_2_. After 18 h, cells were weaned off FBS with DMEM without phenol red, containing 0.2% bovine serum albumin (BSA). and then cells were treated with MPE-298 or oxLDL at 37 °C in 5% CO_2_ for indicated times. At the end of the incubation time, cells were washed with phosphate-buffered saline (PBS) and fixed with paraformaldehyde (1.5% in PBS). Fixed cells were permeabilized with PBS-2% BSA-0.05% saponin buffer for 20 min and stained for EEA1, Rab7, Rab11, and Lamp1 antigens overnight at 4 °C. The next day, the cells were incubated with secondary antibody Alexa-fluor^®^594 anti-rabbit IgG. Pearson’s correlation coefficient for colocalization of CD36 with endocytic markers was assessed using the plugin JACoP (just another colocalization plugin) from ImageJ/Fiji version 1.54k software.

### 2.4. Labeling MPE-298 with ATTO-465-NHS-Dye

A solution of MPE-298 (200 μg) in phosphate buffer (15 mM, pH 6.5) was added to a solution of 150 μg of ATTO-465 NHS ester dissolved in dimethyl sulfoxide (DMSO). The solution was first incubated at room temperature for 2 h and then overnight at 4 °C. The reaction was stopped by adding 0.1% trifluoroacetic acid (TFA)/H_2_O to a final volume of 1 mL. The coupled peptide was isolated by reverse-phase HPLC. The ATTO-465-MPE-298 product was validated for molecular weight by liquid chromatography mass spectrometry (LC/MS-MS).

### 2.5. MPE-298 Internalization and Subcellular Localization

RAW 264.7 cells (ATCC) were seeded at 125,000 cells per well in a 96-well plate and allowed to adhere overnight in Roswell Park Memorial Institute Medium (RPMI, Wisent Inc, Saint-Jean-Baptiste, QC, Canada) supplemented with 10% FBS and 1% penicillin/streptomycin at 37 °C in 5% CO_2_. The cells were weaned off FBS for 2 h in RPMI-25mM Hepes, supplemented with 1% penicillin/streptomycin and 0.2% BSA. The kinetics of ligand internalization (incubation at 37 °C) and plasma membrane bound (incubation at 4 °C) were initiated by adding ATTO-465-MPE-298 at different time points. After incubation, the cells were washed 3 times with cold PBS to determine the total fluorescence and the cell surface bounds or with sodium acetate pH 4 buffer at 4 °C to assess the internalized fraction, followed by lysis in 0.5N NaOH/0.05% sodium dodecyl sulfate (SDS). For the characterization of the endocytosis pathways elicited by MPE-298, cells were pretreated with specific inhibitors 30 min before adding the fluorescent peptide as the tracer. Briefly, after being weaned off FBS, the cells were preincubated at 37 °C in 5% CO_2_ with cytochalasin D (2 μg/mL), as an inhibitor of actin polymerization, and with PP1 (3 μg/mL) and PP2 (3 μg/mL), as inhibitors of Src kinases. After 30 min of incubation, ATTO-465-MPE-298 was added for an additional 15 min. The fluorescence was analyzed with excitation/emission wavelengths at 510/580 nm using a BioTek Synergy2 (Agilent, Santa Clara, CA, USA)microplate reader.

For intra-cellular localization of the fluorescent tracer, RAW264.7 cells were transfected with CellLight^TM^ late endosomes-RFP (Rab7-RFP) or lysosomes-RFP (Lamp1-RFP) according to the manufacturer’s instructions. Twenty-four hours after transfection, cells were starved for 2 h and treated with MPE-298 for 10 and 30 min.

### 2.6. Bioluminescence Resonance Energy Transfer (BRET) Biosensor Constructs

For the construction of mouse CD36-RlucII, CD36 ORF was amplified from pCMV3-mCD36-GFPSpark using the 5′-primer NheI-CD36-Forward: CTATAGGGAGACCCAAGCTGGCTAGCATGGGATGTGATCGGAACTGTGGG and 3′-primer HindIII-CD36-Reverse: TTGCTGGTCATGGTGGCGGGAAGCTTACCCCCTTTCACATTCTTGGATTTG primers to introduce NheI and HindIII digestion sites sequences. The final product was subcloned in-frame into NheI/HindIII of pcDNA3.1/hygromycin (+) plasmid containing the RlucII [19]. The rGFP-CAAX plasmid, constructed as previously described [19], was kindly provided by Dr. Stephane Laporte.

### 2.7. Cell Transfection

For the BRET assay, J774A.1 cells were seeded in a 96-well plate (white, flat bottom) at a density of 35,000 cells/well in DMEM supplemented with 10% FBS and 1% penicillin/streptomycin. Cells were grown at 37 °C in 5% CO_2_ and 90% humidity. The next day, the culture medium was changed to DMEM 10% FBS and 0.2% penicillin/streptomycin, supplemented with 5-azacytidine (1 μM) for 2 h. Cells were transiently transfected using a mixed solution containing mCD36-RlucII plasmid (25.6 ng), rGFP-CAAX plasmid (31.7 ng), and pcDNA3.1(+) for a total of 80 ng cDNA/well. TransIT-X2^®^ reagent was used at a cDNA-to-reagent ratio of 1 μg to 4 μL. Twenty-four hours later, cells were starved for 2 h in Tyrode’s buffer (140 mM NaCl, 2.7 mM KCl, 1 mM CaCl_2_, 12 mM NaHCO_3_, 5.6 mM D-glucose, 0.5 mM MgCl_2_, 0.37 mM NaH_2_PO_4_, and 25 mM HEPES, pH 7.4) supplemented with 0.01% BSA. Luminescence was induced with Prolume Purple (1.5 μM final) for 2 min in Tyrode’s buffer. Luminescence signal was measured using a Tecan Spark Multimode microplate reader (Tecan Trading AG, Männedorf, Switzerlnd) in the BRET2 mode with filter sets of 420/70 nm and 515/30 nm for detecting RlucII and rGFP signals, respectively. The BRET ratio was determined as follows: [(intensity emitted by the rGFP/intensity emitted by the Rluc2) − (intensity emitted by the rGFP/intensity emitted by the Rluc2 for the CD36-RLuc2 control cells)].

For kinetic study, transiently co-transfected J774A.1 cells were incubated at different times with MPE-298 (100 nM) or oxLDL (25 μg/mL). In experimental protocols involving pharmacological inhibitors, cells transiently co-transfected were pretreated with PP1 (3 μg/mL), cytochalasin D (2 μg/mL), piceatannol (40 μM), 2-bromopalmitate (100 μM), SSO (100 μM), and BI-0115 (5 μM) for 30 min. CD36 internalization was then induced with MPE-298 and oxLDL for 15 min. For the BRET saturation studies, the cells were transiently transfected with a fixed concentration of mCD36-RlucII plasmid (25.6 ng) and increasing concentrations of rGFP-CAAX plasmid ranging from 0 to 77 ng for 24 h.

### 2.8. Bone-Marrow-Derived Monocytes/Macrophages Isolation and Treatment

Male mice were housed in the university animal facility and maintained under a 12 h/12 h light/dark cycle. All experiments involving the use of animals were approved by the institutional ethics committee (#23-034) and performed in accordance with the Canadian Council on Animal Care guidelines for the use of experimental animals. Mice aged 8 to 12 weeks old were euthanized with CO_2_ overdose and exsanguinated. Bone-marrow-derived monocytes (BMDM) were isolated and differentiated as previously described [16].

Briefly, bone marrow from femurs and tibias of 8–12-week-old C57BL/6 wild-type (WT) and CD36 knockout (CD36-KO) mice was suspended in DMEM /10% FBS, and cells were cultured for 7 days in the presence of rmM-CSF (40 ng/mL) then washed after 7 days and stimulated for 48 h with LPS (100 ng/mL) and interferon gamma (IFNϒ, 20 ng/mL) for M1 phenotype differentiation. Following 2 h starvation, M1 BMDMs were treated or not for 10 min with MPE-298 (100 nM), oxLDL (25 μg/mL), or the combination of MPE-298 and oxLDL. For the studies involving the LOX-1 inhibitor, cells were preincubated with BI-0115 for 30 min prior to treatment with MPE-298 or oxLDL.

### 2.9. Mitochondrial and Plasma Membrane Isolation and Western Blotting

Murine RAW264.7 cells or M1 BMDMs were seeded in 6-well plates. The next day, cells were starved for 4 h and treated with MPE-298 (100 nM) or oxLDL (25 μg/mL) for the indicated times. Cells were washed with cold PBS, then lysed for 30 min in ice-cold radioimmunoprecipitation assay buffer (RIPA) (150 mM NaCl, 50 mM Tris-HCl, 1% Triton X-100, 0.2% SDS, 50 mM NaF, and 2 mM EDTA, pH 7.4) containing protease and phosphatase inhibitors (Pierce Biotechnology, Waltham, MA, USA). Cell lysates were centrifuged at 15,000× *g* for 20 min at 4 °C.

For mitochondrial and plasma membrane isolation, cells were harvested and incubated in isolation buffer (250 mM sucrose, 250 μg/mL digitonin, 1 mM EDTA, and 50 mM Tris, pH 7.4) containing protease and phosphatase inhibitor cocktails with constant shaking for 10 min. Cells were then disrupted with 15 strokes of a 25-gauge syringe needle. Cell debris and nuclei were removed by centrifugation at 1000× *g* for 5 min (4 °C). The supernatant was centrifuged at 15,000× *g* for 20 min (4 °C). The pellets (mitochondria-enriched fractions) were resuspended in RIPA buffer. The resulting supernatants were centrifuged at 100,000× *g* for 1 h. The pellets (membranes) were resuspended in RIPA, and the supernatants considered as cytosolic fractions were concentrated by Microcon centrifugal filters (Sigma-Millipore, St. Louis, MO, USA).

The protein concentration of supernatants was determined by a bicinchoninic acid (BCA) assay (Pierce Biotechnology, Waltham, MA, USA). Equal amounts of protein extracts were separated on 4–12% SDS–polyacrylamide gels and transferred to polyvinylidene difluoride (PVDF) membranes (Bio-Rad Laboratories, Hercules, CA, USA) for immunoblotting. Membranes were blotted with primary antibodies p47phox and Rac1/2/3 for phosphorylated Syk (Tyr348), as well as SRC family kinases (Tyr416), Lyn (Tyr397), JNK (Thr183/Tyr185), and p66Shc (Ser36). Antibody against β-actin was used as an internal control and antibody against TOM20 was used as the internal control for mitochondria. Immunoblotted bands were detected by enhanced chemiluminescence with West Femto chemiluminescent substrate (Thermo Scientific, Waltham, MA, USA) using a ChemiDoc MP Imaging System (Bio-Rad Laboratories, Hercules, CA, USA). Image analysis was performed using Image Lab 6.1 software (Bio-Rad Laboratories, Hercules, CA, USA).

### 2.10. Mitochondrial Reactive Oxygen Species (mtROS) and Loss of Membrane Potential Measurements

RAW264.7 cells (17,000 cells/well) or M1 BMDM (60,000 cells/well) in complete medium (RPMI 1640 supplemented with 10% FBS and 1% penicillin/streptomycin), were seeded in 96-well tissue culture plates and incubated overnight at 37 °C in 5% CO_2_. On the day of experimentation, cells were starved in RPMI medium supplemented with 1% penicillin/streptomycin and 0.2% BSA for 1 h. Cells were first pretreated with MPE-298 for 1 h and stimulated or not with oxLDL for 4 h. In other experiments, cells were preincubated with inhibitors as indicated in the figure legends prior to MPE-298 incubation and oxLDL stimulation. After the stimulation period, cells were washed with Hanks’ balanced salt solution (HBSS, Wisent Inc.) and incubated with a mixture of MitoSOX Red (5 μM), a mitochondrial superoxide indicator, and Hoechst-33342 (4 μM), a nuclear staining dye, for 20 min at 37 °C. The fluorescence intensities were analyzed using a Synergy2 (BioTek) microplate reader with the following filter sets: excitation/emission at 510/580 nm for MitoSox and 350/461 nm for Hoechst-33342. Fluorescence intensity of MitoSox was normalized by fluorescence intensity of Hoescht-33342.

For the measurement of the mitochondrial membrane potential (ΔΨM), treated cells were incubated in HBSS with 5,5′,6,6′-tetrachloro-1,1′,3,3′-tetraethylbenzimidazolylcarbocyanine iodide (JC-1, 5 μM), a membrane-permeable cationic dye (Invitrogen-Life Technologies, Carlsbad, CA, USA). After 20 min, cells were extensively washed with HBSS. Fluorescence from aggregated JC-1 was measured with an excitation wavelength of 550 nm and emission wavelength of 600 nm, and fluorescence from JC-1 monomer was detected with an excitation wavelength of 485 nm and emission wavelength of 535 nm. The ΔΨM was determined from the ratio of intensities at 600 nm and 535 nm.

### 2.11. CCL2 Measurement

RAW264.7 cells were seeded at a density of 150,000/well in 48-well plates. The next day, the cells were weaned for 4 h in DMEM supplemented with 0.2% BSA, exposed to MPE-298 or oxLDL or the combination of MPE-298 and oxLDL, and incubated at 37 °C in 5% CO_2_ for 16 h. After the incubation period, media were collected and assessed for CCL2 chemokine using a mouse ELISA detection kit (Invitrogen-Life Technologies, Carlsbad, CA, USA) following the manufacturer’s instructions. For experiments using inhibitors, the cells were pre-exposed to SSO for 30 min prior to stimulation with MPE-298 or oxLDL.

### 2.12. Cellular Viability

Cellular viability was assessed using a Cell Counting Kit-8 test (CCK-8, Sigma-Millipore), with tetrazolium salt for measuring the cellular dehydrogenase in living cells. RAW264.7 cells (7500 cells/well) were cultured overnight in a 96-well plate in DMEM supplemented with 10% FBS and 1% penicillin/streptomycin. The cells were then weaned for 2 h in DMEM supplemented with 0.2% BSA, exposed to different concentrations of MPE-298 or oxLDL and incubated at 37 °C in 5% CO_2_ for 24 h. After the incubation period, the cellular viability was assessed following the manufacturer’s protocol.

### 2.13. Statistical Analysis

Statistical analyses were performed using GraphPad Prism 7.0 software (GraphPad Software, Boston, MA, USA). One-way and two-way analyses of variance (ANOVA) were performed. For experiments using a one-way ANOVA statistical analysis, Dunnett’s comparison post-test was performed. For experiments using a two-way ANOVA statistical analysis, Tukey’s multiple comparison post-test was performed. Experimental data are presented as the mean ± standard error of the mean (SEM). A *p*-value lower than 0.05 was considered statistically significant.

## 3. Results

### 3.1. Internalization of MPE-298 in RAW264.7 and J774A.1 Macrophage Cell Lines

**Figure 1:**
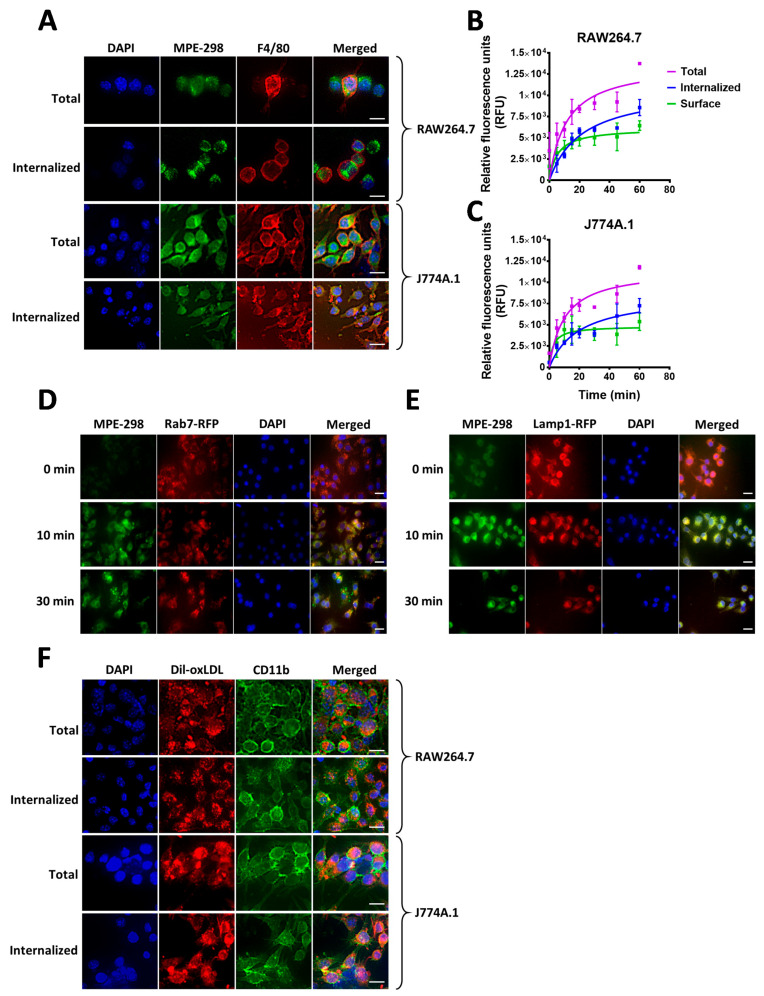
Intracellular localization of MPE-298 following its internalization in macrophages. (**A**) Representative immunofluorescence images of fixed RAW264.7 and J774A.1 cells treated with fluorescent ATTO-465-MPE298. (**B**,**C**) Relative fluorescent units (RFU) of ATT-465-MPE-298 kinetics of internalization after PBS (total) or acid wash (internalized) of the cells in RAW264.7 and J774A.1 cells, respectively. (**D**,**E**) Intracellular localization of ATTO-465-MPE-298 with late-endosomal and lysosomal markers, respectively. (**F**) Uptake of Dil-oxLDL in RAW264.7-and J774A.1 cell lines. Scale bar size: 20 µm.

Endocytosis and trafficking of fluorescently-tagged oxLDL (Dil-oxLDL) and of ATTO-465-conjugated MPE-298 azapeptide were studied in RAW264.7 and J774A.1 macrophage-like cell lines. After an acid wash to remove excess tracer, internalized fluorescent azapeptide was detected in both cell lines (Figure 1A). The kinetic profiles showed a rapid uptake of about 60% of the total fluorescence within 10 min after exposure of both RAW264.7 and J774A.1 cell lines to fluorescent-labeled MPE-298 (Figure 1B,C). After 10 min of incubation, the fluorescent-labeled MPE-298 was found to colocalize with late endosome (Rab7) and lysosome markers (Lamp1) in RAW264.7 cells (Figure 1D,E). Similarly to the internalization of labeled MPE-298, fluorescently tagged oxLDL was found to be internalized within 25 min of incubation with RAW264.7 and J774A.1 cell lines (Figure 1F).

**Figure 2:**
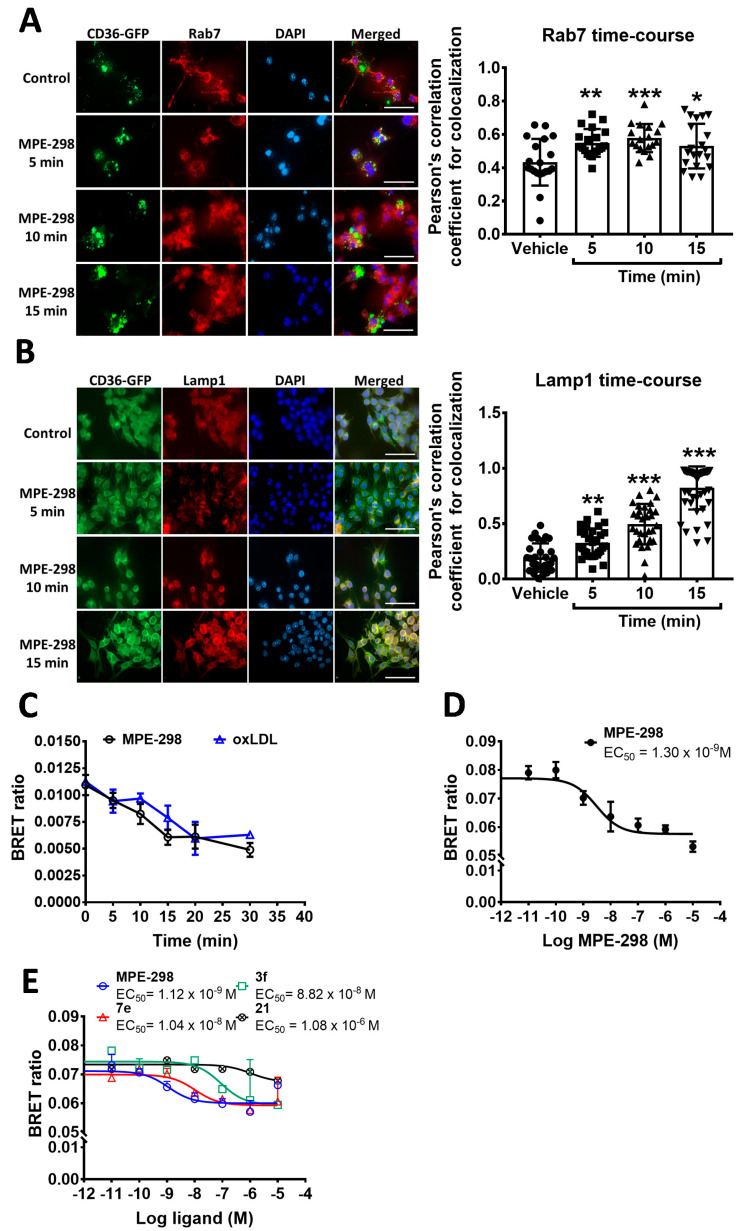
Intracellular localization of the CD36-MPE-298 complex following its internalization in macrophages. (**A**,**B**) mCD36-GFPspark-transfected RAW264.7 cells were stained with Rab7 (late endosome) and Lamp1 (lysosome) markers, respectively. Pearson’s correlation coefficients are presented as a function of time. Data are presented as the mean ± SEM. A one-way ANOVA test with Dunnett’s comparison post-test was performed. * *p* < 0.05, ** *p* < 0.01, and *** *p* < 0.005 vs. vehicle. (**C**) Kinetics of CD36 internalization in a BRET-based assay in J774A.1 cells transiently co-expressing mCD36-RlucII and rGFP-CAAX in the presence of MPE-298 (100 nM) or oxLDL (25 μg/mL) at different times. (**D**) Dose–response of MPE-298 in a BRET-based CD36 internalization assay in co-transfected J774A.1 macrophages. (**E**) Dose–response curves of MPE-298 analogs 3f, 7e, and 21 using a BRET-based CD36 internalization in co-transfected J774A.1 cells. Scale bar size: 50 µm.

Intracellular trafficking of the internalized complex composed of MPE-298 and CD36 was also monitored in J774A.1 cells, which were transfected with mouse CD36 tagged with green fluorescent protein (mCD36-GFP). After treatment with MPE-298, transfected J774A.1 cells exhibited a rapid colocalization (after 5 min) of CD36 with the late endosome marker Rab7 (Figure 2A) and the lysosome marker Lamp1 (Figure 2B). Localization of CD36 in the late endosome plateaued at 5 min, and slightly decreased after 15 min of incubation (Figure 2A). The localization of CD36 in the lysosome was time-dependent (Figure 2B). Treatment with MPE-298 also caused a rapid colocalization of CD36 with the early endosome marker, EEA1 (Figure S1A). However, no increase in colocalization was observed with Rab11, a marker of recycling endosomes (Figure S1B). In contrast to the treatment with MPE-298, the exposure of transfected J774A.1 cells to oxLDL resulted in the rapid localization of CD36 in early endosomes (after 5 min) with slight colocalization with Rab7 after 15 min (Figure S2A,B).

The ability of MPE-298 to trigger CD36 endocytosis was further validated employing a sensitive bioluminescence resonance energy transfer (BRET) approach featuring transient co-transfection of J774A.1 macrophages with mCD36-Rluc2 (donor) and rGFP-CAAX (acceptor). We found that the BRET signal ratio of the acceptor/donor expression was saturable when cells were transfected at a fixed donor concentration (Figure S3A). The obtained average ratio values for BRETmax and BRET50 were 0.15 and 0.7, respectively. With a fixed donor concentration (25.6 ng), BRET50 was estimated at 18 ng of the acceptor. For the BRET experiment, we used the BRET75 ratio, which was calculated to correspond to 31.7 ng of the acceptor. We next monitored for endocytosis of CD36 following incubation with MPE-298 and oxLDL. Both ligands induced CD36 internalization, as indicated by the decreased BRET signal ratio (Figure 2C). The decrease in the BRET signal was found to be time-dependent and 15 min incubation was the optimal time for studying CD36 endocytosis using the BRET technique.

The sensitivity of the BRET approach in macrophages was further illustrated by performing a dose–response curve of MPE-298 in co-transfected J774A.1 cells (Figure 2D). Incubation of transfected cells with different concentrations of MPE-298 triggered endocytosis of CD36 in a concentration-dependent manner with an EC_50_ of 1.3 nM. In parallel, increasing concentrations of oxLDL-induced endocytosis of CD36 in a dose-dependent manner, with an EC_50_ of 1.45 μg/mL (Figure S3B). Furthermore, the azapeptide-binding affinity correlated with the potential to induce endocytosis of CD36 using the BRET assay. The CD36-binding affinities, previously reported using competitive binding assays, of azapeptide analogs 7e (4.75 μM), 3f (22.8 μM) and 21 (>1 mM) [17,18], correlated with the rates of endocytosis of their respective CD36 complexes, as follows: EC_50_ of 10.4 nM, 882 nM, and 1080 nM, respectively (Figure 2E). All together, these data confirm the selectivity of CD36 ligands in promoting the internalization of the azapeptide/CD36 complex.

### 3.2. MPE-298 Induces Phosphorylation of Syk and Src Kinases in RAW264.7 Macrophages

**Figure 3:**
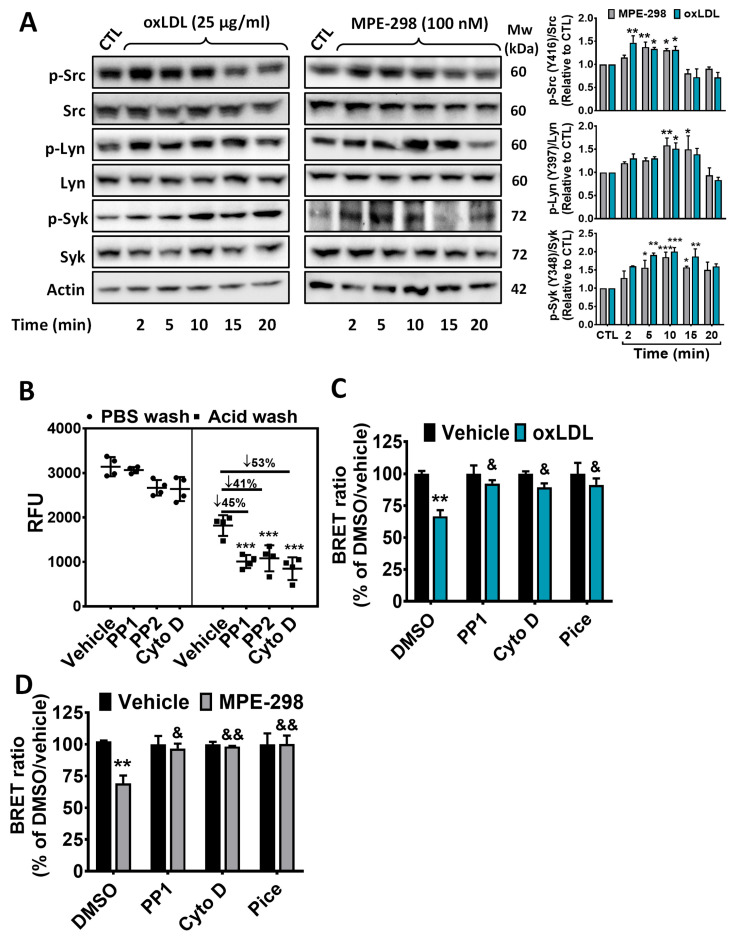
CD36-mediated MPE-298 and oxLDL intracellular signaling pathways in macrophages cell lines. (**A**) Representative Western blots of kinetic studies of total and phosphorylated Src (Tyr 416), Lyn (Tyr 397), and Syk (Tyr 348) kinases in RAW264.7 macrophages treated with MPE-298 or oxLDL. Relative quantification values are expressed as the ratio of phosphorylated/total protein normalized to the control. Data are expressed as the mean ± SEM of the fold change over the control (CTL) (n = 3 different experiments). A one-way ANOVA test with Dunnett’s comparison post-test was performed. * *p* < 0.05, ** *p* < 0.01, and *** *p* < 0.001 vs. CTL. (**B**) Internalization of ATTO-465-MPE-298 (500 nM) in the presence of PP1, PP2 (Src inhibitors), or cytochalasin D (Cyto D, inhibitor of actin polymerization). Data are presented as the mean relative fluorescence units (RFUs) ± SEM (n = 3 experiments performed in triplicate). (**C**,**D**) BRET-based assay of oxLDL- or MPE-298-induced CD36 endocytosis in mCD36-RlucII/rGFP-CAAX co-transfected J774A.1 cells exposed to PP1, Cyto D, and piceatannol (Pice) (n = 3 independent experiments performed in triplicate). Data are expressed as the percentage of DMSO/vehicle. Data are presented as mean ± SEM. A two-way ANOVA test with Tukey’s multiple comparison post-test was performed. ** *p* < 0.01 and *** *p* < 0.001 vs. DMSO/vehicle. & *p* < 0.05 and && *p* < 0.01 vs. DMSO/oxLDL.

Endocytosis of oxLDL by CD36 has been reported to activate intracellular signaling pathways, including those of the Src family protein kinases and spleen tyrosine kinase (Syk) [2]. The activation of these kinases by MPE-298 was measured by phosphorylation at tyrosine residues Tyr416, Tyr397, and Tyr348 of Src, Lyn, and Syk kinases, respectively, in RAW264.7 cells (Figure 3A). Upon treatment with MPE-298, phosphorylation of Src and Lyn was detected by as early as 2 min and lasted for 10 min in the case of the former. Phosphorylation of the Syk kinase at Tyr348 was gradually enhanced for 10 min after exposure to MPE-298. Moreover, within 2 min of the treatment of RAW264.7 cells with oxLDL, the phosphorylation of Src, Lyn, and Syk kinases was enhanced and sustained for up to 10 min (Figure 3A).

Next, pharmacological inhibitors were used to validate the involvement of Src kinases and the actin network in macrophage uptake of the complex between MPE-298 and CD36. The uptake of fluorescently labeled MPE-298 into RAW264.7 cells decreased by 45%, 41%, and 53%, respectively, after pretreatment with the Src kinase inhibitors PP1 and PP2, as well as with the inhibitor of actin polymerization cytochalasin D (Figure 3B). Next, the effects of the inhibitors of Src and Syk kinase signaling on the endocytosis of CD36 after treatment with MPE-298 and oxLDL for 15 min was assessed in transfected J774A.1 cells. As shown in Figure 3C,D, BRET signals were reduced by 30% and 33% in the cells exposed to oxLDL and MPE-298, respectively. Following pretreatment with PP1, cytochalasin D and the Syk inhibitor piceatannol, the effects of both ligands on the BRET signal (Figure 3C,D) were completely abolished. These results suggest that both MPE-298 and oxLDL share common and conserved core components for the endocytosis of CD36.

### 3.3. MPE-298 Inhibits oxLDL-Triggered Inflammation and Mitochondrial Oxidative Stress in RAW264.7 Cells

**Figure 4:**
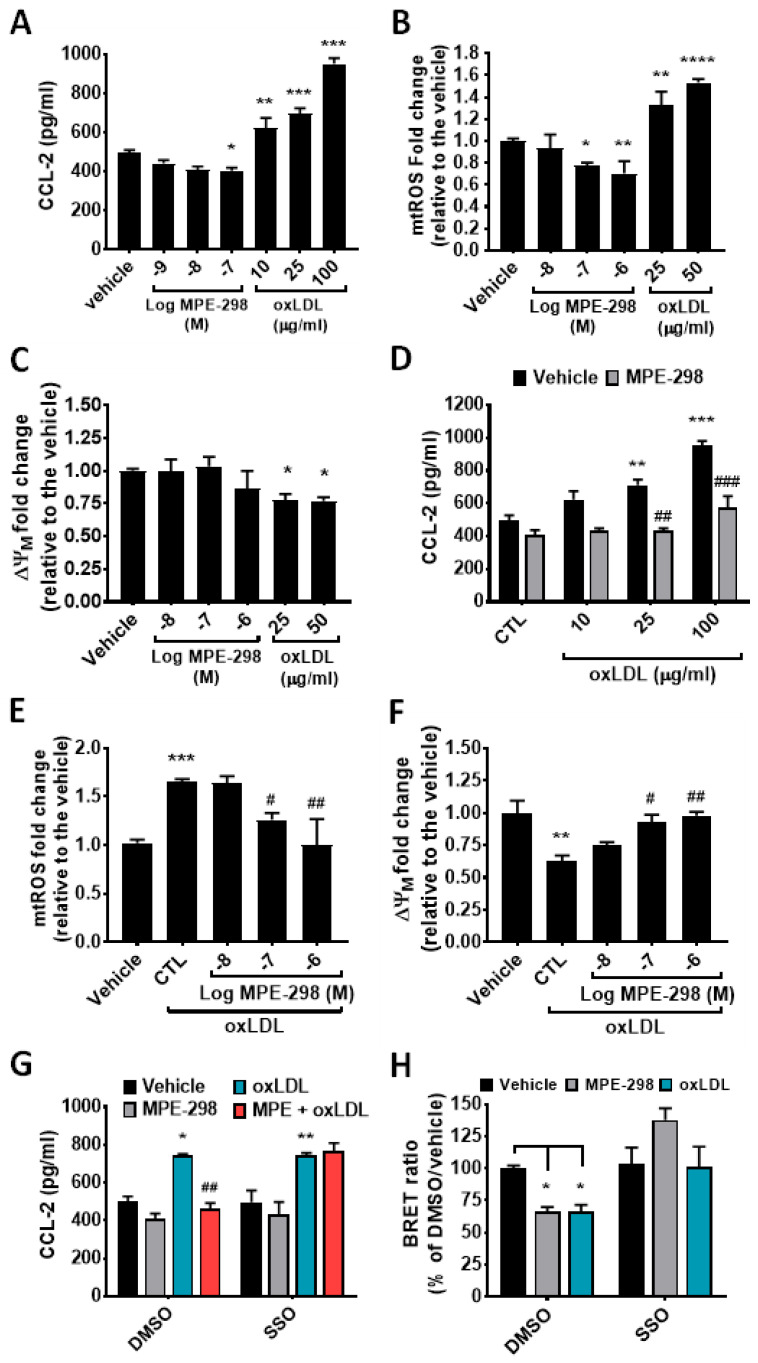
MPE-298 modulatory effects on mitochondrial oxidative stress elicited by oxLDL in murine RAW264.7 cell lines macrophages. (**A**) CCL2 secretion in the supernatants of cells exposed to different concentrations of MPE-298 or oxLDL for 24 h. Data are presented as the mean ± SEM (n = 3 experiments performed in triplicate). (**B**) Mitochondrial reactive oxygen species production (mtROS) and (**C**) mitochondrial membrane potential (ΔΨM) in RAW264.7 cells treated with different concentrations of MPE-298 or oxLDL for 4 h. (**D**) Inhibitory effect of MPE-298 (100 nM) on oxLDL-induced CCL2 secretion in cells stimulated with different concentrations of oxLDL for 24 h. (**E**,**F**) MPE-298 dose–response inhibition of oxLDL (25 μg/mL)-induced mtROS production and ΔΨM loss, respectively. (**G**) CD36-mediated inhibitory effect of MPE-298 on CCL2 secretion in the presence or absence of the CD36 inhibitor, SSO (100 μM). (**H**) BRET-based assay of oxLDL- and MPE-298-induced CD36 endocytosis in the absence or presence of SSO in mCD36-RlucII/rGFP-CAAX co-transfected J774A.1 cells. Data in (**A**,**D**,**G**) are presented as mean ± SEM; data in (**B**,**C**,**E**,**F**,**H**) are expressed as the percentage of vehicle and presented as mean ± SEM. n = 3 independent experiments, each conducted in triplicate. In (**A**–**C**,**E**,**F**), a one-way ANOVA test with Dunnett’s comparison post-test was performed. * *p* < 0.05, ** *p* < 0.01, *** *p* < 0.001, and **** *p* < 0.0001 vs. vehicle. # *p* < 0.05 and ## *p* < 0.01 vs. oxLDL/CTL. In (**D**,**G**,**H**) a two-way ANOVA test with Tukey’s multiple comparison post-test was performed. * *p* < 0.05, ** *p* < 0.01, and *** *p* < 0.001 vs. control (CTL)/vehicle or DMSO/vehicle; ## *p* < 0.01 and ### *p* < 0.001 vs. oxLDL/vehicle or oxLDL/DMSO.

The anti-inflammatory properties of MPE-298 as CD36 modulators were previously reported to implicate co-receptor interactions with the TLRs complex of activated macrophages [17,18]. In contrast, scavenger receptor binding of oxLDL stimulated NADPH oxidase activity and promoted oxidative stress, ROS production, and inflammation in macrophages [12,20,21]. In defining the optimal experimental conditions for this in vitro study, we found that the macrophage cell viability was significantly influenced at high concentrations of MPE-298 (10 to 100 μM) and oxLDL (≥30 μg/mL) (Figure S4A,B). Furthermore, secretion of the chemokine CCL2 in macrophages was decreased by exposure for 24 h to MPE-298 (1 to 100 nM). In contrast, macrophage exposure to 10, 25, and 100 μg/mL of oxLDL augmented the secretion of CCL2 by 25%, 49%, and 91%, respectively (Figure 4A). The production of mtROS was shown to significantly increase and decrease in a dose-dependent manner upon exposure of RAW264.7 macrophages to oxLDL and MPE-298, respectively, as ascertained using MitoSox Red as the reporter (Figure 4B). Moreover, the exposure of RAW264.7 macrophages to oxLDL and MPE-298 caused a loss and no change, respectively, in the mitochondrial membrane potential (ΔΨM), as assessed with JC-1 (Figure 4C). Finally, pre-exposure of RAW264.7 macrophages to MPE-298 was shown to dose-dependently reverse the effects of oxLDL on CCL2 secretion, mtROS production, and ΔΨM loss (Figure 4D–F).

Next, the involvement of CD36 in the inhibitory activity of MPE-298 on oxLDL-induced inflammation was ascertained with SSO, an irreversible inhibitor of scavenger receptor function [22]. Exposure of RAW264.7 macrophages to oxLDL caused a 50% increase in CCL2 secretion, which was unaltered by preincubation with 100 μM of SSO. Moreover, the effects of MPE-298 on oxLDL-triggered CCL2 release were completely abolished upon pre-exposure to SSO (Figure 4G). Using the BRET approach, exposure to MPE-298 and oxLDL resulted in net losses in the BRET signal of about 31% and 30%, respectively. Moreover, preincubation with SSO completely abolished the losses in the BRET signal induced by MPE-298- and oxLDL-mediated endocytosis of CD36 (Figure 4H). Overall, the use of SSO indicates that the modulatory effects of MPE-298 correlated with the binding and endocytosis of CD36.

### 3.4. MPE-298 Disrupts LOX-1 Receptor-Mediated oxLDL Mitochondrial Oxidative Stress

**Figure 5:**
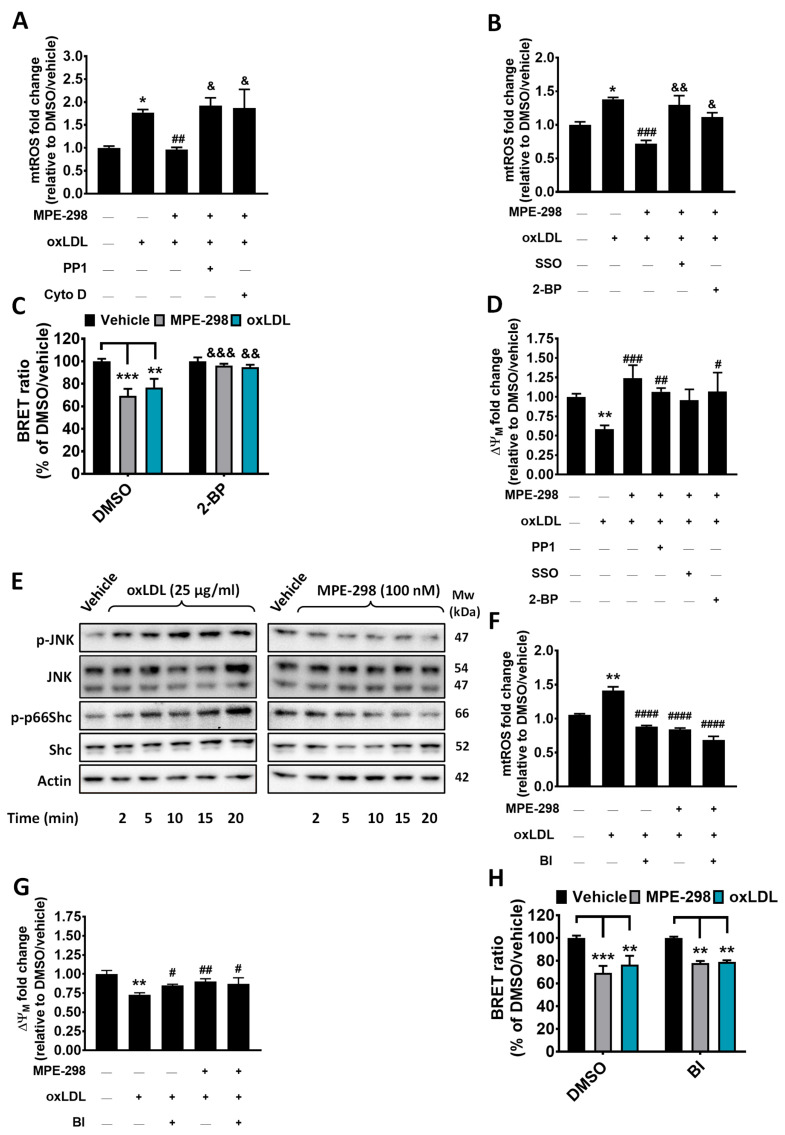
MPE-298 attenuates mitochondrial oxidative stress induced by oxLDL by mitigating LOX-1 signaling pathways in murine RAW264.7 macrophage cells. The assessment of MPE-298’s inhibitory effects on oxLDL-induced mtROS production in the presence or absence of the pharmacological inhibitors of endocytosis (**A**) PP1 (3 μg/mL), cytochalasin D (Cyto D, 2 μg/mL), and (**B**) sulfo-N-succinimidyl oleate (SSO, 100 μM) or 2-bromopalmitate (2-BP, 100 μM). (**C**) Assessment of the BRET-based assay following oxLDL- and MPE-298-induced CD36 endocytosis in mCD36-RlucII/rGFP-CAAX co-transfected J774A.1 cells exposed to 2-BP. (**D**) Effect of MPE-298 inhibition on ox-LDL-induced ΔΨM or the presence of the pharmacological inhibitors PP1, Cyto D, SSO, or 2-BP. (**E**) Western blots of the kinetic studies of the total and phosphorylated JNK (Thr183/Tyr185) and p66Shc (Ser36) in RAW264.7 macrophages treated with MPE-298 (100 nM) and oxLDL (25 μg/mL). Representative blots of 3 experiments. (**F**,**G**) Inhibitory effect of MPE-298 on oxLDL-induced mtROS production and ΔΨM loss, respectively, in the presence or absence of the pharmacological inhibitor of LOX-1, BI-0115 (BI, 5 μM). (**H**) The BRET-based assay in mCD36-RlucII/rGFP-CAAX co-transfected J774A.1 cells exposed to oxLDL and MPE-298 in the absence or presence of BI. All data are expressed as the mean ± SEM of three independent experiments each conducted in triplicate. Data are expressed either as the percentage of vehicle (**C**,**H**) or as the fold change relative to the vehicle and presented as the mean ± SEM. (**A**,**B**,**D**,**F**,**G**) A one-way ANOVA test with Dunnett’s comparison post-test was performed. * *p* < 0.05 and ** *p* < 0.01 vs. vehicle (no-treatment); # *p* < 0.05, ## *p* < 0.01, ### *p* < 0.001 and #### *p* < 0.0001 vs. oxLDL-treated; & *p* < 0.05 and && *p* < 0.01 vs. MPE-298/oxLDL. (**C**,**H**) A two-way ANOVA test with Tukey’s multiple comparison post-test was performed. ** *p* < 0.01 and *** *p* < 0.001 vs. DMSO/vehicle; && *p* < 0.01 and &&& *p* < 0.001 2-BP vs. DMSO.

Selective pharmacological inhibitors were next employed to interrogate the CD36-dependent mechanisms by which MPE-298 antagonized oxLDL-induced mtROS production and mitochondrial membrane potential. The use of the CD36 inhibitor SSO, the kinase inhibitor PP1, and the inhibitor of actin polymerization cytochalasin D all blocked the protective effect of MPE-298, which had mitigated the 1.8-fold increase in mtROS caused by oxLDL stimulation of RAW264.7 macrophages (Figure 5A,B). Considering that localization of CD36 in the plasma membrane and its internalization are linked to palmitoylation and depalmitoylation, respectively, of *N*- and *C*-terminal cysteine residues by palmitoyl–acyltransferase and acyl protein thioesterase enzymes [23,24,25], 2-bromopalmitate (2-BP) was employed as a non-selective palmitoylation inhibitor known to block acyl protein thioesterase activity [26]. The exposure of RAW264.7 cells to 2-BP (100 μM) blocked MPE-298 from inhibiting oxLDL-elicited mtROS production (Figure 5B). On the other hand, neither SSO nor PP1 and 2-BP altered the 1.4-fold mtROS production elicited by oxLDL in RAW264.7 cells (Figure S4C). Employing the BRET approach in transfected J774A.1 cells, 2-BP was shown to abolish the 31% and 23% losses in the BRET signal caused by MPE-298 and oxLDL, respectively (Figure 5C). The pretreatment of RAW264.7 cells with MPE-298 prevented the 1.4-fold loss of the mitochondria membrane potential elicited by oxLDL (Figure 5D). Pre-exposure of RAW264.7 cells to the inhibitors SSO, PP1, and 2-BP did not block MPE-298 from reversing oxLDL-elicited mitochondrial membrane depolarization (Figure 5D). However, the inhibitors SSO, PP1, cytochalasin D, and 2-BP all prevented the effect of oxLDL on mitochondrial membrane depolarization in RAW264.7 cells (Figure S4D).

Other signaling pathways mediating the activity of MPE-298 on oxLDL-induced mitochondrial stress and inflammation were investigated in RAW264.7 cells. It is known that oxLDL activates the MAP kinases c-Jun *N*-terminal kinases (JNK)-1 and -2 in macrophages [27] and endothelial cells [28]. This activation influences the regulation of mitochondrial redox status [28], which is under the control of p66Shc, an adaptor protein of the ShcA family [29]. The influences of oxLDL and MPE-298 on JNK and p66Shc phosphorylation were probed by Western blot analyses of RAW264.7 macrophages. Cells exposed to oxLDL exhibited a time-dependent increase in JNK and p66Shc phosphorylation (Figure 5E). In contrast, JNK and p66Shc phosphorylation decreased in cells treated with MPE-298. Activation of JNK and the production of ROS have been reported to be linked to oxLDL binding of lectin-like oxidized low-density lipoprotein receptor-1 (LOX-1) on endothelial cells [30,31] and macrophages [11]. The role of LOX-1 in the CD36-mediated activity of MPE-298 was investigated using BI-0115, a small-molecule inhibitor that blocks oxLDL uptake and prevents LOX-1-mediated signaling [32]. Although BI-0115 (5 μM) mitigated the mtROS production and mitochondrial membrane potential depolarization elicited by oxLDL, blocking LOX-1 had no effect on the inhibitory activities of MPE-298 (Figure 5F,G). Moreover, BI-0115 exposure did not alter the effects of MPE-298 and oxLDL on CD36 endocytosis (Figure 5H). Overall, internalization of the complex between MPE-298 and CD36 appears to modulate the LOX-1-mediated mitochondrial oxidative stress elicited by oxLDL in macrophages.

### 3.5. MPE-298 Blocks oxLDL-Induced JNK and p66Shc Activation in M1 Bone-Marrow-Derived Macrophages

**Figure 6:**
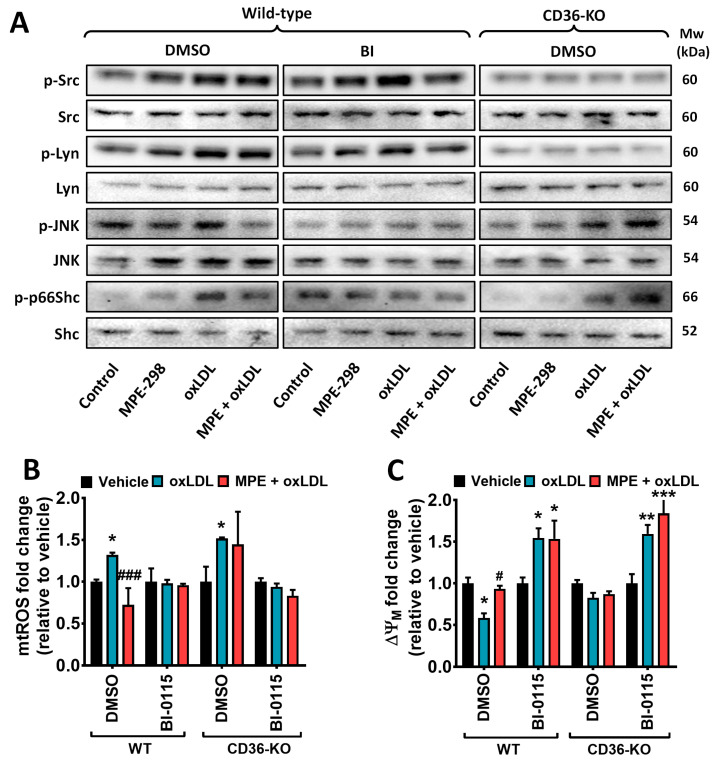
MPE-298 attenuates oxLDL-induced mitochondrial damage in a CD36-dependent manner in primary M1 bone-marrow-derived macrophages (BMDM). (**A**) Western blots of the total and phosphorylated Src (Tyr416), Lyn (Tyr397), and JNK (Thr183/Tyr185) kinases and p66Shc (Ser36) M1-phenotype BMDMs. Differentiated cells from wild-type (WT) and CD36-KO mice were preincubated with or without the LOX-1 inhibitor BI-0115 (5 μM), followed by treatment with MPE-298 (100 nM) or oxLDL (25 μg/mL), or with a combination of MPE-298 and oxLDL. Representative blots of two experiments. The effect of MPE-298 on oxLDL-induced (**B**) mtROS production and (**C**) ΔΨM loss in the absence or presence of BI-0115. The experiments are expressed as the mean ± SEM of two independent experiments each conducted in triplicate. Data were assessed as the fold change relative to vehicle and are presented as the mean ± SEM. Two-way ANOVA test with Tukey’s multiple comparison post-test was performed. * *p* < 0.05, ** *p* < 0.01, and *** *p* < 0.001 vs. vehicle; # *p* < 0.05 and ### *p* < 0.001 vs. oxLDL-treated cells.

The signaling mechanism of the complex between MPE-298 and CD36 was further investigated using bone-marrow-derived macrophages (BMDMs) isolated from WT and CD36-KO mice. After treatment with a combination of LPS and IFNγ to induce an M1 phenotype, BMDMs were exposed to MPE-298 and oxLDL alone, as well as in sequence with or without the LOX-1 inhibitor BI-0115. As observed in RAW264.7 cells, increased phosphorylation at Tyr416 and Tyr394 of the Src and Lyn kinases, respectively, was observed employing MPE-298 and oxLDL alone and in combination in treated BMDMs from WT mice. The inhibition of LOX-1 by BI-0115 did not affect the ability of MPE-298 or oxLDL to induce Src and Lyn phosphorylation. However, in BMDMs from CD36KO mice, treatment with MPE and oxLDL failed to induce the phosphorylation of both kinases (Figure 6A). Considering the opposite modulatory effects on JNK and p66Shc exhibited by MPE-298 and oxLDL in RAW264.7 macrophages, the effects of combined treatment and the role of LOX-1 in the activation of these proteins was evaluated in BMDMs. Compared to untreated cells, a 10 min treatment with MPE-298 or oxLDL caused a decrease and an increase in JNK phosphorylation, respectively, in BMDM from WT mice. In combination, MPE-298 and oxLDL decreased JNK phosphorylation compared to incubation with oxLDL alone. Phosphorylation at Ser36 of p66Shc was unchanged over 10 min of incubation with MPE-298 alone but was found to increase with oxLDL incubation alone. The combined MPE-298 and oxLDL exposure decreased this phosphorylation (Figure 6A). Inhibition of LOX-1 with BI-0115 prevented oxLDL from inducing the phosphorylation of JNK and p66Shc. In contrast, CD36 knockdown did not prevent oxLDL-induced phosphorylation of JNK and p66Shc, whereas the inhibitory effect of MPE-298 on both the JNK and p66Shc phosphorylation elicited by oxLDL was not evidenced, as observed in WT BMDMs (Figure 6A).

The respective roles of CD36 and LOX-1 were delineated using M1-polarized BMDM from WT and CD36-KO mice to ascertain the effects on mtROS and membrane potential depolarization of oxLDL alone and in combination with MPE-298. Incubation of WT- and CD36-KO-BMDM with oxLDL resulted in a 1.3-fold increase in mtROS production. Following pretreatment with MPE-298, this mtROS increase was completely blocked in WT, although remained unaffected in CD36-KO-BMDM (Figure 6B). Moreover, preincubation with MPE-298 attenuated the 0.4-fold decrease in membrane potential depolarization caused by oxLDL in BMDM from WT compared to untreated cells (Figure 6C). Blocking LOX-1 with BI-0115 in BMDM from WT exposed to oxLDL resulted in a 1.6-fold increase in ΔΨM compared to the control vehicle-treated cells and remained unaltered following preincubation with MPE-298. In CD36-KO-BMDM, the mitochondria membrane potential was affected neither by oxLDL nor MPE-298, but it increased by 1.6-fold upon inhibition of LOX-1 following preincubation with MPE-298, as observed in WT-BMDM (Figure 6C).

### 3.6. MPE-298 Disrupts LOX-1 Receptor-Mediated oxLDL Activation of NADPH Oxidase 2 in RAW264.7 Cells

**Figure 7:**
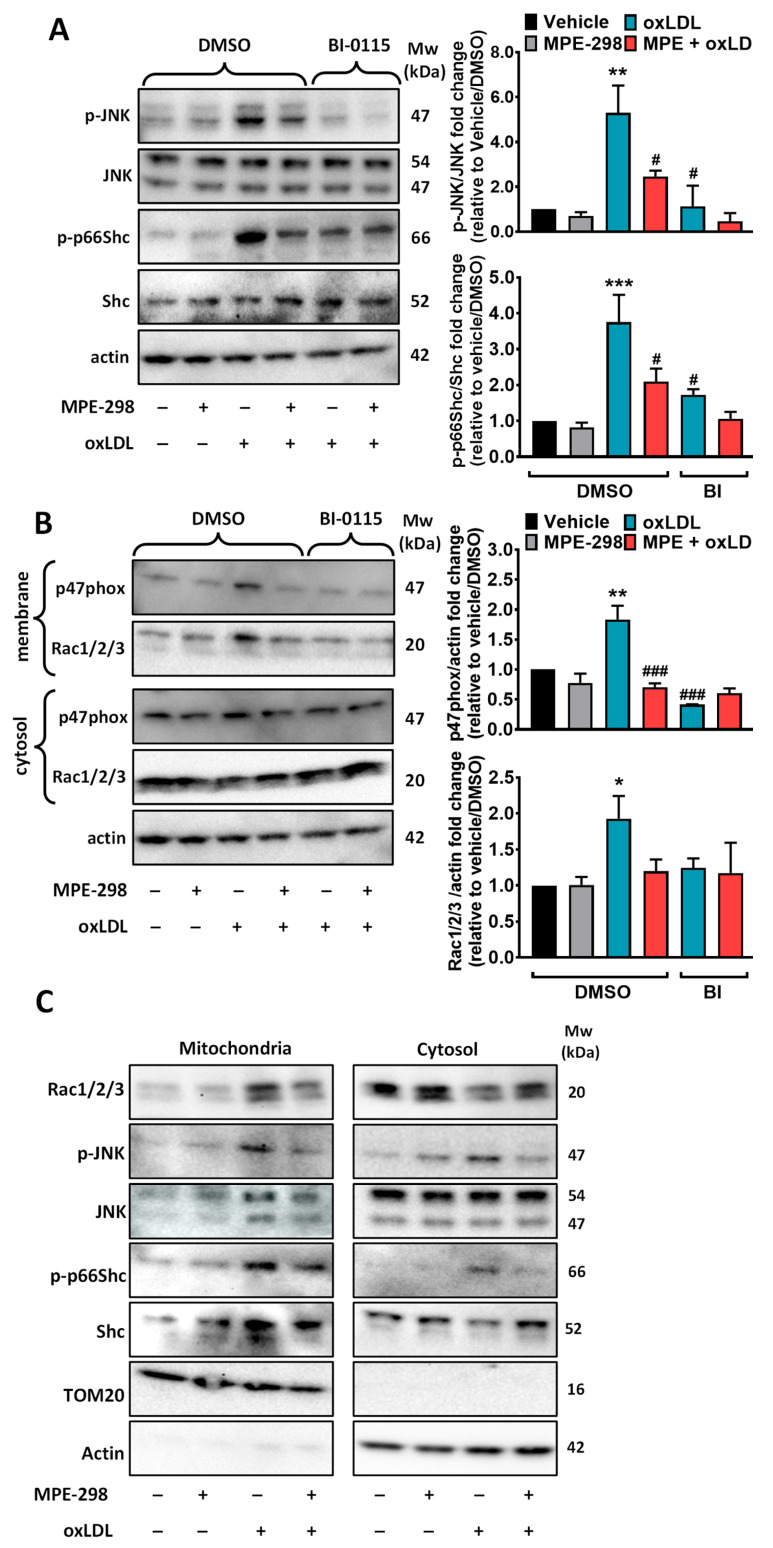
MPE-298 inhibits LOX-1-mediated NOX2 activation by oxLDL in the plasma membrane. of RAW264.7 cells (**A**) Western blots of the cell lysates of MPE-298- or oxLDL-treated cells in the presence or absence of the LOX-1 inhibitor BI-0115 (5 μM) using antibodies against the total and phosphorylated JNK (Thr183/Tyr185) and p66Shc (Ser36). Representative blots of three experiments. Data are expressed as the fold change relative to the vehicle and presented as the mean ± SEM of the ratio of phosphorylated/total protein. (**B**) Western blots of the plasma membrane and cytosolic fractions from treated RAW264.7 cells using antibodies for p47phox and RAC1/2/3. Representative blots of three independent experiments. Data are expressed as the fold change over the vehicle and presented as the mean ± SEM. A one-way ANOVA test with Dunnett’s comparison post-test was performed. * *p* < 0.05; ** *p* < 0.01, and *** *p* < 0.001 vs. vehicle; # *p* < 0.05, ### *p* < 0.001 vs. oxLDL-treated. (**C**) Western blots of mitochondria and cytosolic membrane fractions from cells treated with MPE-298 (100 nM) or oxLDL (25 μg/mL). (n = 3 independent experiments).

The role of LOX-1 in the mitigating effect of oxLDL-induced inflammation caused by the complex between MPE-298 and CD36 was ascertained in RAW264.7 cells using BI-0115. Incubation of RAW264.7 cells for 10 min with oxLDL and MPE-298 led to a 5.3-fold increase and no effect, respectively, on JNK phosphorylation (Figure 7A). In contrast, preincubation of RAW264.7 cells with MPE-298 prior to stimulation with oxLDL completely blocked JNK phosphorylation (Figure 7A). Furthermore, BI-0115 attenuated the oxLDL-induced phosphorylation of JNK independent of MPE-298. Similarly, the 3.8-fold increase in phosphorylation of p66Shc induced by oxLDL in RAW264.7 cells was blocked by preincubation with MPE-298 (Figure 7A). Treatment with BI-0115 decreased the induction of p66Shc phosphorylation in RAW264.7 macrophages. Incubation of RAW264.7 cells with the CD36 inhibitor SSO did not prevent oxLDL-induced JNK and p66Shc phosphorylation but abolished the effects of MPE-298 (Figure S5). These results suggest that the complex between MPE-298 and CD36 modulates LOX-1-mediated signaling pathways induced by oxLDL.

LOX-1 signaling has been associated with the activation of the NADPH oxidase 2 (NOX2) core complex receptor in the plasma membrane of macrophages [11]. For NOX2 activation, subunits p47phox, p67phox, and p40phox are recruited and interact with p91phox/NOX2 and p22phox in the plasma membrane to assemble the NOX2 core complex. Translocation of p47phox facilitates the recruitment of Rac1/2/3 GTPase, leading to ROS production [33,34]. Furthermore, Rac1 was reported to play a pivotal role in the phosphorylation of p66Shc [35]. Considering that the complex between MPE-298 and CD36 inactivated p66Shc, the consequent effects on NOX2 core activation were examined in macrophages. Plasma membranes were isolated from RAW264.7 macrophages, which were treated with MPE-298 or oxLDL with and without the BI0115 inhibitor. The recruitment of p47phox and Rac1/2/3 to the membrane increased 1.8-fold and 1.9-fold with oxLDL but was unaffected by MPE-298 (Figure 7B). The blocking of LOX-1 with BI0115, as well as preincubation with MPE-298, inhibited the recruitment of both p47phox and Rac1/2/3 elicited by oxLDL in the plasma membrane of RAW264.7 macrophages (Figure 7B). Oxidative stress has been proposed to induce the activation of JNK and Rac1/2/3, which translocate into mitochondria, where they activate and stabilize p66Shc, leading to the amplification of ROS production [35,36,37,38]. The effects of oxLDL and MPE-298 on the activation of p66Shc and the translocation of Rac1/2/3 and JNK into mitochondria were evaluated in RAW264.7 macrophages. Exposure to oxLDL resulted in the translocation and activation of p66Shc, as indicated by phosphorylation at Ser36 (Figure 7C). Treatment with MPE-298 prevented phosphorylation of p66Shc. Compared to the vehicle-treated cells, incubation with oxLDL was observed to cause translocation of Rac1/2/3 into mitochondria and increased the phosphorylation of JNK, both of which were blocked by preincubation with MPE-298 (Figure 7C).

## 4. Discussion

Among the pattern recognition receptors (PRRs) involved in macrophage recognition of oxLDL, CD36 plays a critical role in the onset and development of chronic inflammatory pathologies, such as atherosclerosis and degenerative retinal diseases, namely, age-related macular degeneration. Macrophage activation by oxLDL is especially critical for foam cell formation and stimulation of the immune response [39]. In addition, the ability of CD36 to form heterocomplexes with TLRs allows for the initiation of cell- and context-dependent transcriptional pathways integrating specific TLR immune responses [7]. Moreover, CD36 forms TLR-independent signaling complexes that activate transcriptional and non-transcriptional pathways during the inflammatory response [1,2]. Probing the interplay between oxLDL and PRR, we have elucidated the mechanism of a novel intervention for mitigating macrophage-driven inflammatory pathology. The small, six-amino-acid synthetic azapeptide MPE-298, which is a selective CD36 ligand, was found to regulate the response of macrophages to oxidative stress and inflammatory stimuli. Upon binding, the complex between MPE-298 and CD36 undergoes rapid endocytosis by activating the Src kinase family members Lyn and Syk in RAW264.7 macrophages in a time-dependent manner. After rapid internalization, the complex colocalized in the endosome and lysosome compartments. We further discovered that the internalized complex blocked oxLDL-induced LOX-1-mediated recruitment of Rac1/2/3 and p47phox in the plasma membrane, thereby preventing activation of NOX2. Moreover, the internalized complex inhibited oxLDL promotion of inflammation and mitochondrial damage including pathways featuring activation of JNK and p66Shc by phosphorylation.

Using a novel BRET approach, the endocytosis of CD36 and the binding affinity of its ligands were correlated. We also found that endocytosis of the complex between CD36 and MPE-298 was impaired by exposure to the inhibitors of Src, Syk, and actin polymerization in macrophages. Accordingly, modified lipoproteins and fatty acids were reported to induce CD36 endocytosis by processes implicating the activation of Src kinase family members, including Lyn and Syk, in the membrane [1,2,40]. In macrophages exposed to oxLDL, the Src and Syk kinase pathways were involved in the activation of actin and vimentin filaments, which serve in the internalization of CD36 and the accumulation of cholesterol crystals in lysosomes [2,41]. Furthermore, an anti-CD36-specific antibody reduced cholesterol accumulation in oxLDL-exposed macrophages and dampened mediators of downstream signaling [2,41]. Moreover, our data inferred that CD36 endocytosis induced by MPE-298 might involve de-palmitoylation of the receptor by acyl-protein thioesterase (APT) 1 and APT2. Indeed, MPE-298-induced internalization of CD36 in macrophages was blocked in the presence of 2-bromopalmitate (2-BP), which has been suggested to inhibit APT1/2 activity by binding in a non-covalent manner [26,42]. De-palmitoylation of CD36 by acyl-protein thioesterase (APT) 1 and APT2 was reported to favor endocytosis, as suggested in the uptake of fatty acids and modified lipoproteins [23,40]. Members of the Asp-His-His-Cys motif-containing enzyme family, such as DHHC5 and DHHC6, exhibit palmitoyl acyltransferase activity and protect CD36 at the membrane by countering de-palmitoylation [23,40,43]. It was reported that Lyn kinase phosphorylation inactivated DHHC5/6 and facilitated Syk kinase recruitment with consequent CD36 endocytosis [23,40]. Accordingly, macrophage exposure to MPE-298 rapidly activated Lyn and Syk kinases and may, thereby, trigger DHHC5/6 inhibition to promote CD36 endocytosis.

Molecular mechanisms regarding the mitigating effects of MPE-298 on oxLDL-induced inflammation in macrophages were also elucidated. Although both MPE-298 and oxLDL triggered the internalization of CD36 in macrophages, MPE-298 inhibited JNK and p66Shc, which, in contrast, were activated by oxLDL. Exposure of M1-phenotype BMDMs and RAW264.7 cells to MPE-298 prevented oxLDL-induced secretion of the inflammatory mediator CCL-2, production of mtROS, and depolarization of the mitochondrial membrane. Inhibition of MPE-298 triggered the endocytosis of CD36 by knocking down or blocking the receptor, as well as by inhibiting the downstream signaling cascades, all completely abolished the mitigating effects of the azapeptide on inflammation and oxidative stress. Endocytosis of the complex between MPE-298 and CD36 prevented the ability of oxLDL to induce the recruitment of p47phox and Rac1/2/3 GTPase at the membrane in a LOX-1-dependent manner, and, consequently, curbed the formation of the NADPH oxidase 2 (NOX2) complex. Mitochondrial damage and CCL-2 secretion in RAW264.7 cells were shown to be induced by oxLDL and dependent upon LOX-1, as the use of the BI-0115 compound decreased mtROS and chemokine release. Rac1/2/3 translocation to the NOX2 complex at the membrane, as well as mitochondrial translocation and activation of p66Shc, JNK, and Rac1/2/3 upon oxLDL exposure, all were prevented by MPE-298. Our findings are in line with previous reports showing that the silencing of LOX-1 with siRNA in oxLDL-treated primary macrophages and in RAW264.7 cells downregulated the expression of NOX2, Rac1/2/3, and p47phox and impaired ROS production, in addition to deactivation of extracellular signal-regulated kinase (ERK) and JNK [11]. Like CD36, LOX-1 is a scavenger receptor that binds oxLDL and mediates downstream signaling pathways in many cell types, such as endothelial cells, fibroblasts, cardiomyocytes, and macrophages [9,11]. In human aortic endothelial cells, incubation with oxLDL led to the activation of p66Shc by phosphorylation at Ser36 in a LOX-1-, p47phox-, Rac1-, and JNK-dependent manner [10]. Serving as an ROS sensor that translocates to the mitochondria, p66Shc is required for amplification of mtROS, membrane depolarization, and release of cytochrome-c induced by oxidative stress [38]. Aortae from ApoE^−/−^/p66Shc^−/−^-deficient mice fed a high-fat diet exhibited fewer atherosclerotic lesions and lipid-laden macrophage foam cells, as well as reduced oxidative stress, compared to their ApoE^−/−^/p66Shc^+/+^ counterparts [44]. In parallel to the activation of p66Shc, mitochondrial translocation of JNK and Rac1 has been reported to enhance apoptosis and inflammation in murine T cells [45] and alveolar macrophages [46], as well as neuronal oxidative stress [47]. Our data show that preventive exposure to MPE-298 prior to stimulation with oxLDL reduced the translocation of activated JNK and Rac1. Highlighting the relevance of the mitochondrial localization of JNK in regulating inflammation, the inhibitor of apoptosis signal-regulating kinase 1 (ASK1), selonsertib, alleviated mitochondrial damage and inflammation in macrophages from a mouse model of acute liver failure induced by treatment with LPS and D-galactosamine [48]. Translocation of Rac1 to the mitochondria has been reported to promote the generation of mitochondrial hydrogen peroxide in macrophages [49], and the overexpression of active Rac1 in human umbilical vein endothelial cells resulted in mitochondrial oxidative stress [50]. In addition, the polyphenolic salvianolic acid from Danshen root was shown to bind CD36 [51] and attenuate oxLDL-induced LOX-1-mediated activation of NOX2 and JNK, as well as mtROS generation, by modulating levels of the mechanotransducer Rho-associated coiled-coil-containing protein kinase (ROCK) 1 in endothelial cells maintained under high-glucose conditions [52]. A limitation of the present study lies in its primarily focus on macrophages derived from differentiated monocytes known to play a central role in the initiation and progression of atherosclerosis. Although future research should also target endothelial cells that express high levels of LOX-1 and CD36 in the recruitment of circulating monocytes in the amplification of the inflammatory response. Moreover, the mitochondrial lipid peroxidation is known to induce mitochondrial oxidative damage through amplification of ROS production. The molecular mechanism by which the internalized MPE298-CD36 modulates mitochondrial lipid peroxidation in mitigating ROS generation will be deciphered in future studies.

## 5. Conclusions

**Figure 8:**
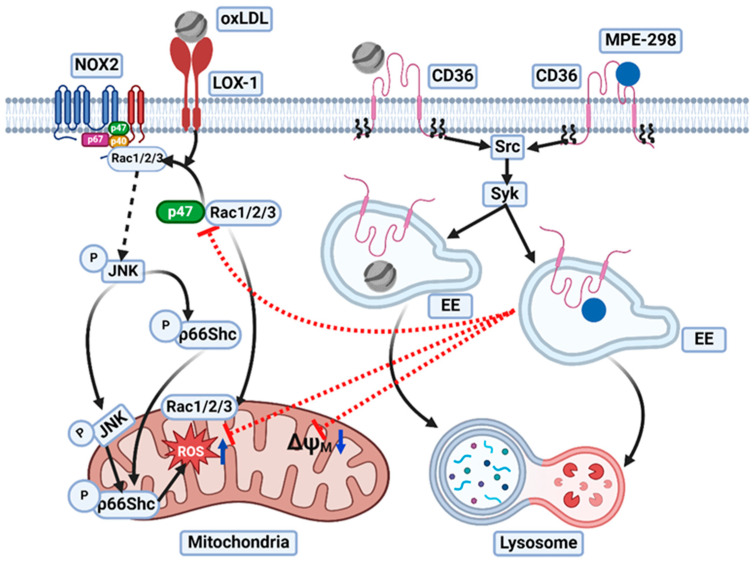
Molecular mechanisms underlying CD36-mediated MPE-298 regulation of oxidative stress induced by oxLDL in macrophages. Binding of azapeptide MPE-298 induces macrophage CD36 endocytosis through the activation of the Src/Syk kinases pathway and the depalmitoylation of the receptor. The internalized MPE-298/CD36 complex prevents oxLDL/LOX-1-mediated recruitment of subunit p47phox and Rac1/2/3 GTPase, which are essential for the formation of the NADPH oxidase 2 (NOX2) complex, disrupting the oxLDL/LOX-1/NOX2-induced activation and translocation of JNK and p66Shc into the mitochondria. This prevents mitochondrial ROS production. ΔΨM: mitochondria membrane potential; EEs: early endosomes; JNK: c-Jun N-terminal protein kinase; LOX-1: lectin-like oxidized low-density lipoprotein receptor 1; NOX2: NADPH oxidase 2; oxLDL: oxidized low-density lipoprotein; p66Shc:Shc: protein 66 Src homology 2 domain (SH2) at C-terminal; ROS: reactive oxygen species; Syk: spleen tyrosine kinase. Created in BioRender. Mulumba, M. (2024) https://BioRender.com/k26k106, (accessed on 28 February 2025).

In conclusion, the CD36-modulating azapeptide MPE-298 has been shown to mitigate the pathology of chronic inflammatory diseases via a dual-molecular mechanism. Acting as a ligand for a membrane co-receptor of the TLR2/6 heterodimer complex, MPE-298 has previously been shown to alter TLR signaling in the activation of inflammatory processes. In the present study, the complex composed of MPE-298 and CD36 has been demonstrated to initiate its internalization into macrophages and to transduce a non-transcriptional signaling pathway. As summarized in Figure 8, this complex was shown to curb oxLDL-induced LOX-1-mediated signaling pathways with subsequent inhibition of NOX2-dependent inflammation, reduction in mitochondrial oxidative stress, and extinction of macrophage-driven inflammatory activity, suggesting its potential in the treatment of chronic inflammatory diseases such as atherosclerosis.

## Data Availability

All uncropped Western blots and datasets generated in this study are available upon request from the corresponding author.

## Supplementary Materials

The following supporting information can be downloaded at: https://www.mdpi.com/article/10.3390/cells14050385/s1, Figure S1. Intracellular disposition of the CD36 complex following its internalization in macrophages after treatment with MPE-298. RAW264.7 cells were transfected with mCD36-GFPspark and treated with MPE-298 (100 nM) for the indicated times; Figure S2. Intracellular disposition of the CD36 complex following its internalization in macrophages after treatment with oxLDL; Figure S3. Dose–response assays of the BRET-based CD36 internalization. Figure S4. Assessment of CD36 blocking with various inhibitors on oxLDL-induced mitochondrial oxidative stress. Figure S5. Western blots of total lysates of treated RAW264.7 cells.
