# Supplementary material for: Selective Azapeptide CD36 Ligand MPE-298 Regulates oxLDL-LOX-1-Mediated Inflammation and Mitochondrial Oxidative Stress in Macrophages
Source: Cells. 2025 Mar 6;14(5):385. doi: 10.3390/cells14050385 (PMC11898605; doi:10.3390/cells14050385)
Supplement: Supplementary file 1 [file cells-14-00385-s001.zip › cells-3314061-supplementary.docx]

**Supplementary Materials**


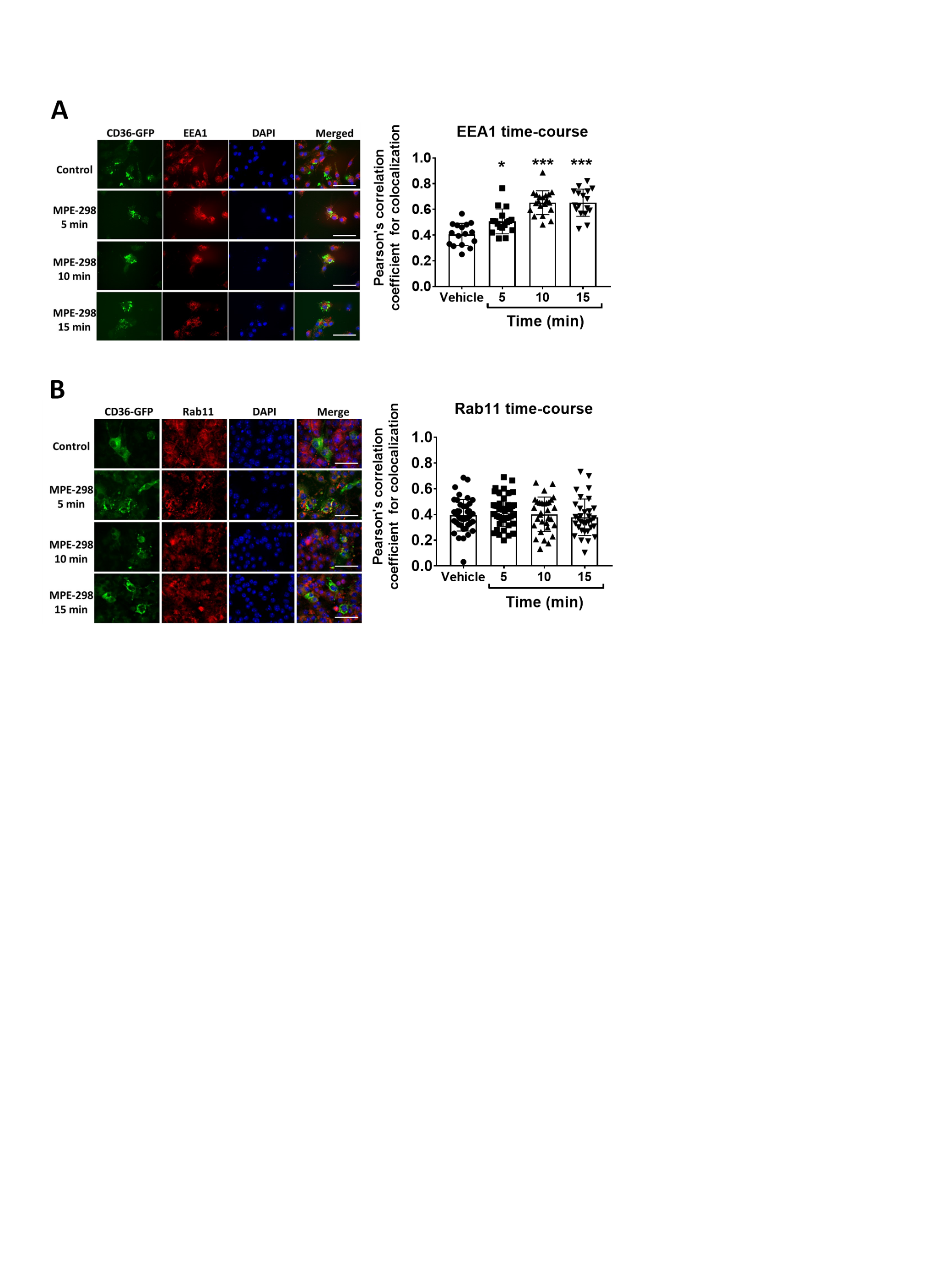


**Figure S1.** Intracellular disposition of CD36 complex following its internalization in macrophages after treatment with MPE-298. RAW264.7 cells were transfected with mCD36-GFPspark and treated with MPE-298 (100 nM) for the indicated times. Representative immunofluorescence images of fixed cells that were stained for an early endosome (**A**) or recycling endosome marker (**B**). Pearson’s correlation coefficients were calculated as described in the Materials and methods section. Data are presented as mean ± SEM. One-way ANOVA test with Dunnett’s comparison post-test was performed. *, p < 0.05; **, p < 0.01 and ***, p < 0.005 vs vehicle. Scale bar size: 50 µm


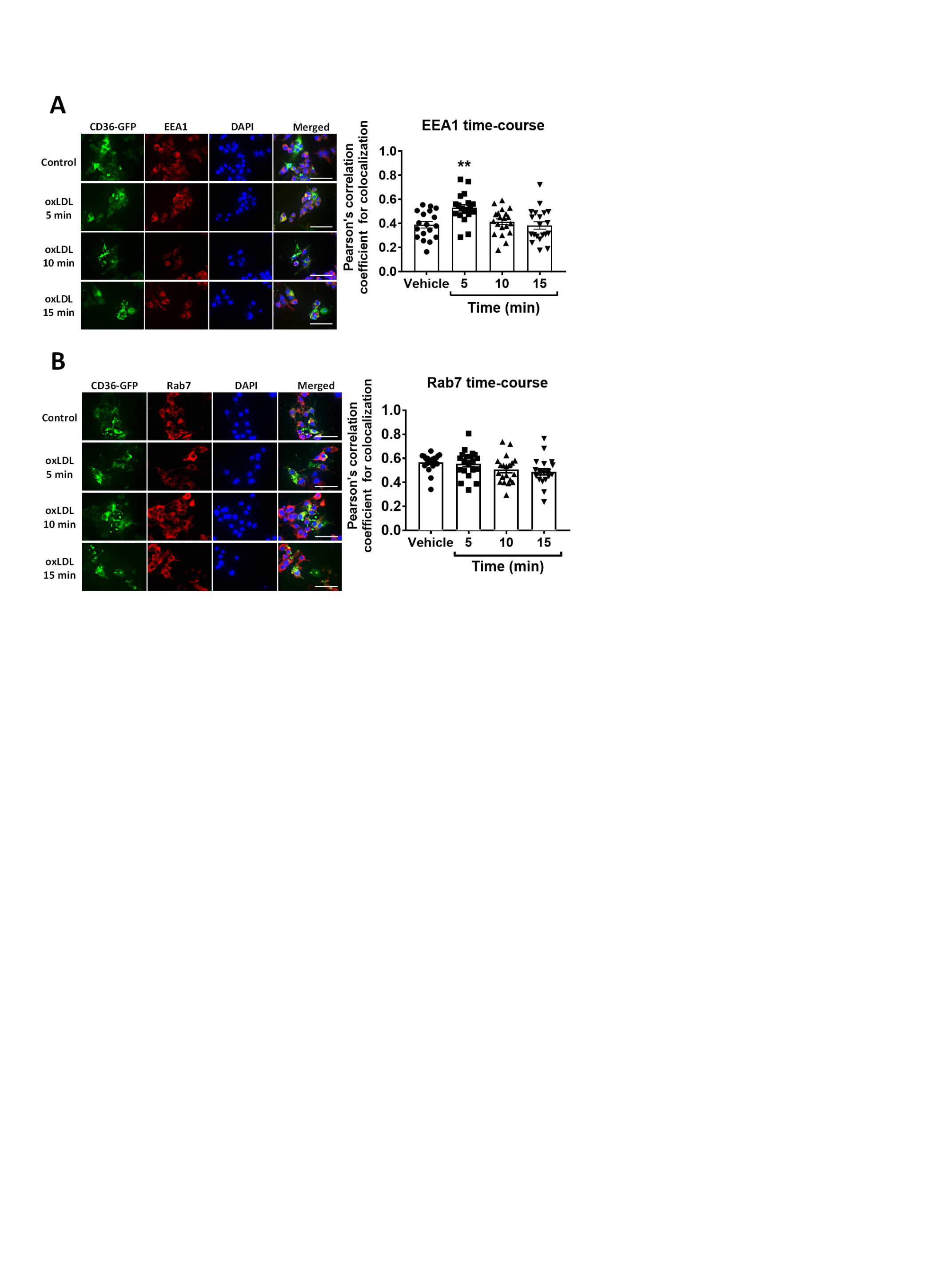


**Figure S2.** Intracellular disposition of CD36 complex following its internalization in macrophages after treatment with oxLDL. J774A.1 cells were transfected with mCD36-GFPspark and treated with oxLDL (25 μg/ml) for the indicated times. Representative immunofluorescence images of fixed cells that were stained with an early endosome (**A**) or lysosome marker (**B**). Pearson’s correlation coefficients were calculated as described in the Materials and methods section. Data are presented as mean ± SEM. One-way ANOVA test with Dunnett’s comparison post-test was performed. **, p < 0.01 vs vehicle. Scale bar size: 50 µm

**
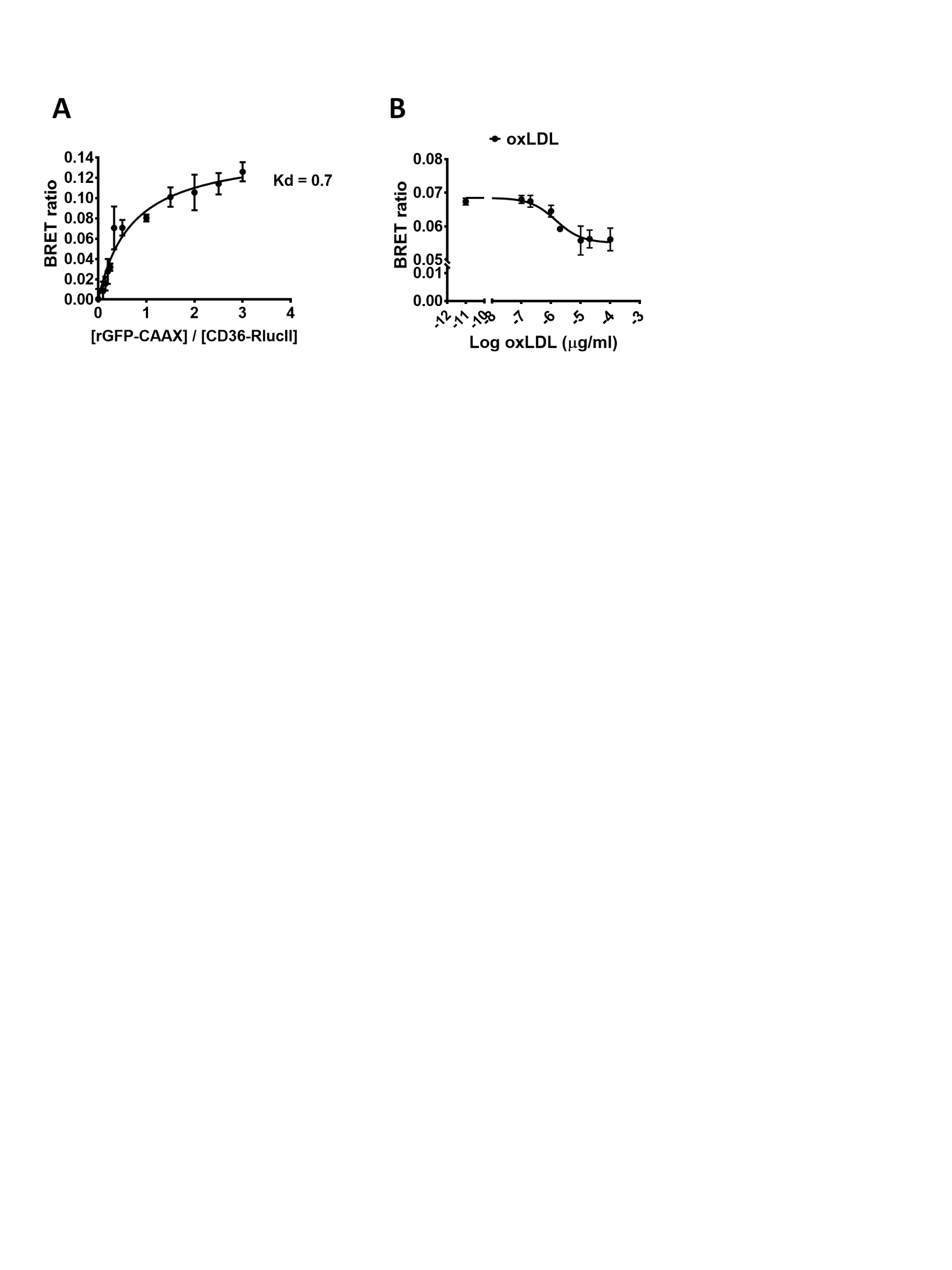
**

**Figure S3.** Dose-response assay of BRET-based CD36 internalization. (**A**) BRET acceptor saturation assay. BRET was measured in J774A.1 macrophage cells transiently co-expressing mCD36-RLuc2 (donor) and rGFP-Caax (acceptor). Co-transfection was performed with increasing amounts of rGFP-Caax plasmid and constant amount of mCD36-RLuc2 plasmid (25.6 ng). Data are presented as mean ± SEM (N = 3). (**B**) Dose-response assay of BRET-based CD36 internalization in J774A.1 macrophages transiently co-expressing mCD36-RLuc2 and rGFP-Caax after 15 min incubation with increasing concentrations of oxLDL.


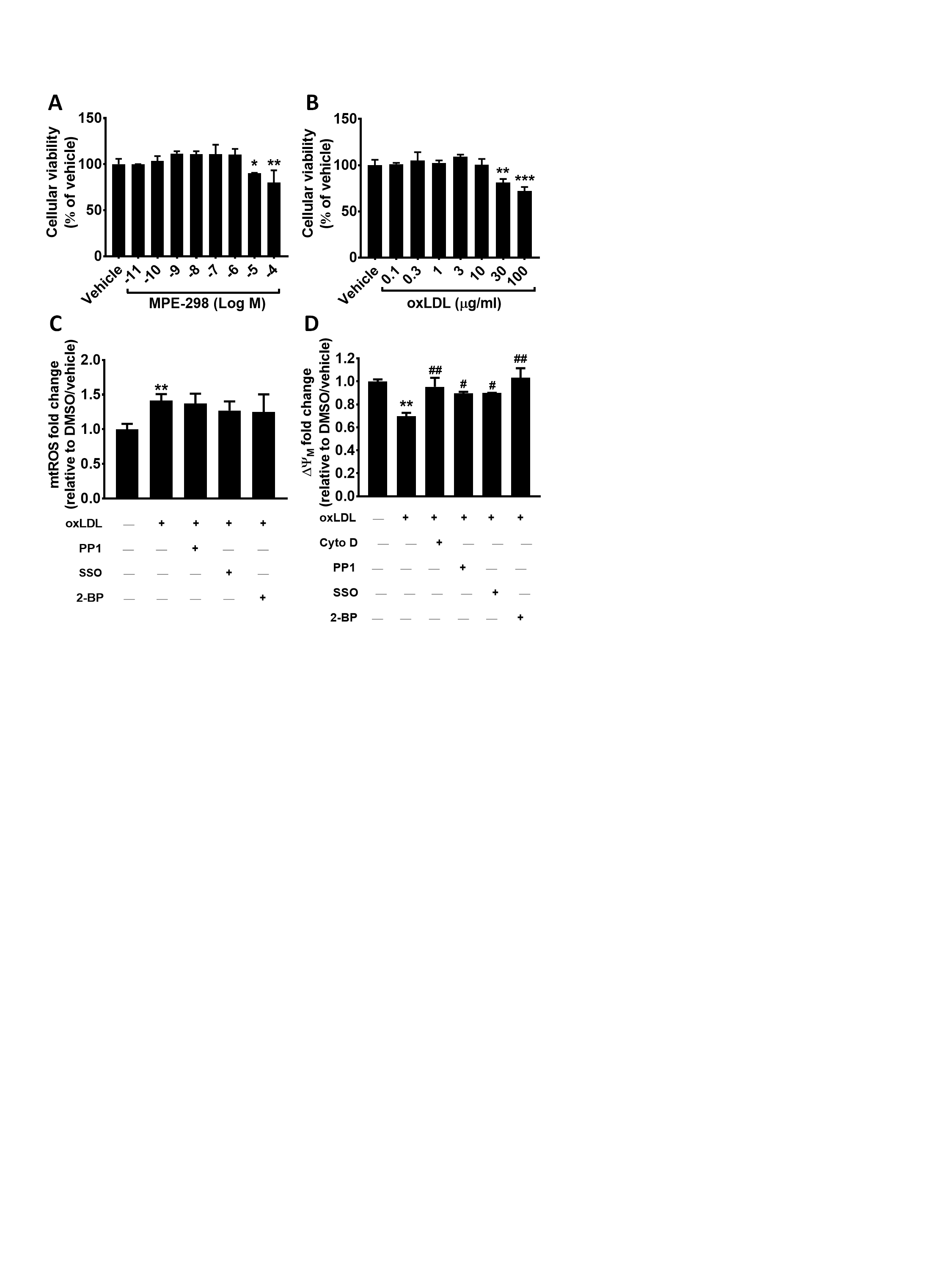


**Figure S4.** Assessment of CD36 blocking with various inhibitors on oxLDL-induced mitochondrial oxidative stress. (**A**) and (**B**) RAW264.7 macrophage cellular viability was assessed with CCK8 after 24 h of treatment with different concentrations of MPE-298 or oxLDL. Assessment oxLDL-induced mtROS production (**C**) and ΔΨM loss (**D**) in the presence of pharmacological inhibitors of CD36 endocytosis PP1 (3 μg/ml), cytochalasin D (Cyto D, 2 μg/ml), sulfo-N-succinimidyl oleate (SSO, 100 μM) and and 2-bromopalmitate (2-BP 100 μM). Following a pre-exposure to PP1, Cyto D, SSO or 2-BP for 30 min, RAW264.7 cells were stimulated with oxLDL (25 μg/ml) for 4 h (n = 3 experiments performed in triplicate). Data are presented as mean ± SEM. One-way ANOVA test with Dunnett’s comparison post-test was performed. **, p < 0.01 vs DMSO/vehicle; #, p < 0.05 and ##, p < 0.01 vs oxLDL-treated.


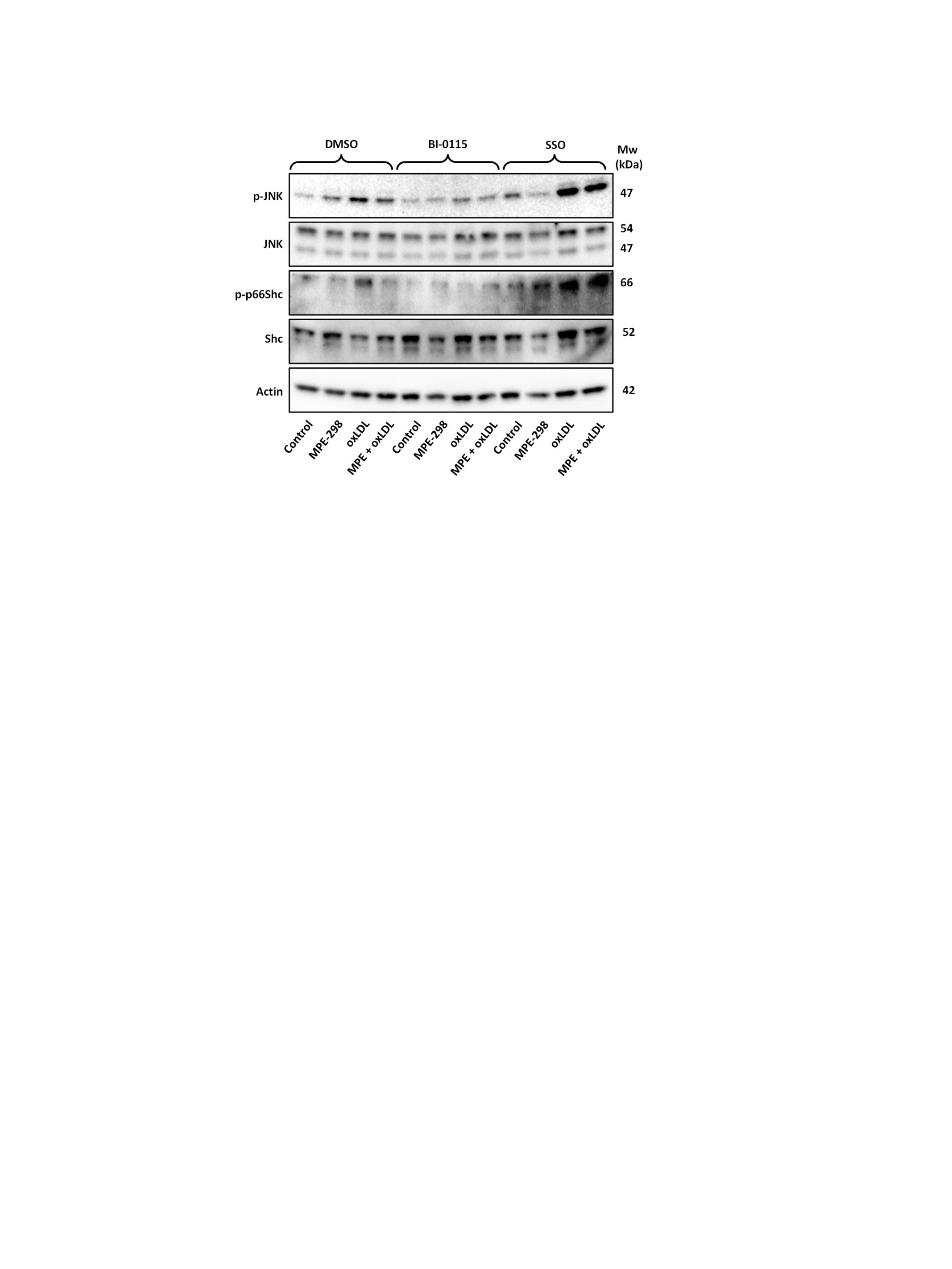


**Figure S5.** Western blots of total lysates of treated RAW264.7 cells. Cells were preincubated with or without the LOX-1 inhibitor, BI-0115 (5 μM) and sulfosuccinimidyl oleate (SSO 100 μM) for 30 min. After incubation, cells were treated with MPE-298 (100 nM) or oxLDL (25 μg/ml) or with the combination of MPE-298 and oxLDL for 10 min. Membranes were blotted for actin and for total and phosphorylated JNK (Thr183/Tyr185) and p66Shc (Ser36). Representative blots of 3 experiments.
